# Development and Validation of an Automated Pipeline for Motor Unit Analysis from HD-sEMG Signals

**DOI:** 10.3390/s26175539

**Published:** 2026-08-31

**Authors:** Maria V. Arteaga, Catalina Alvarado-Rojas, Alain Kaelin-Lang, Daniele Allegri

**Affiliations:** 1 Institute of Systems and Applied Electronics (ISEA), University of Applied Sciences and Arts of Southern Switzerland (SUPSI), 6900 Lugano, Switzerland; maria.arteaga@supsi.ch; 2 Faculty of Biomedical Sciences, Università della Svizzera Italiana, 6900 Lugano, Switzerland; 3 Department of Electronics Engineering, Pontificia Universidad Javeriana, Bogotá 110231, Colombia; catalina_alvarado@javeriana.edu.co; 4 Neurocenter of Southern Switzerland, EOC, 6900 Lugano, Switzerland; 5 Medical Faculty, University of Bern, 3008 Bern, Switzerland

**Keywords:** automated pipeline, blind source separation, high-density surface electromyography, motor unit decomposition, spike-train validation, motor unit conduction velocity, discharge rate, recruitment threshold

## Abstract

High-density surface electromyography (HD-sEMG) enables non-invasive analysis of motor unit (MU) behavior and is crucial in rehabilitation, pathophysiological assessment, and assistive technologies. However, current pipelines still rely on expert intervention, particularly for spike-train editing and channel selection for motor unit conduction velocity (MUCV) estimation. This study developed and validated an automated pipeline for MU analysis from HD-sEMG signals acquired during isometric trapezoidal contractions. The pipeline integrates blind source separation (BSS)-based decomposition, automated source validation and spike-train selection, estimation of discharge rate (DR), recruitment threshold (RT), and automatic channel selection for MUCV. Validation was first performed on a public dataset containing manually edited spike trains from eight operators. The proposed procedure matched 106 of 135 reference MUs with an overall median rate of agreement of 0.98 among matched MUs. Sensitivity analysis supported the robustness of the predefined parameters. The complete pipeline was then evaluated on 276 HD-sEMG signals acquired from the tibialis anterior (TA) muscle of nine healthy volunteers in four sessions using a custom-built acquisition device. The resulting MU parameters were physiologically plausible, and within-session tracking showed high repeatability of MU properties even after electrode repositioning. Validation was limited to healthy volunteers, and further evaluation in clinical populations is needed. These findings support the feasibility of reducing operator dependency while maintaining the quality of HD-sEMG-based MU analysis.

## 1. Introduction

The generation of voluntary movement and force is controlled by the nervous system with the excitation of motor neurons that travel from the motor cortex in the brain to the spinal cord motoneurons, which finally activates the muscles. The motor unit (MU) consists of a motor neuron in the spinal cord and all the muscle fibers it innervates. Action potentials (APs) from the motor neuron produce the contraction of the muscle fibers, and the summation of the electrical activity of a large number of MUs is measured on the surface of the skin as surface electromyography (sEMG) signals [1]. Therefore, analyzing sEMG signals is a crucial non-invasive way to understand neuromuscular function in both physiological and pathological contexts.

Studying the properties of MUs is especially important in contexts where motor function is impaired, such as after neurological injuries (e.g., stroke, spinal cord injury) [2] or in degenerative conditions like Parkinson’s disease [3]. During rehabilitation, longitudinal studies of individual MU parameters [4,5] could provide information about the paresis mechanisms, the progress of the corticospinal muscle rehabilitation, and the development of more efficacious therapeutic interventions. Additionally, MU monitoring could give insights into motor learning mechanisms, including applications to optimize high-level sports performance [4,6] and the design of active prosthetics [7].

Conventional sEMG signals allow only for a global estimation of the muscle activation [8]. In contrast, high-density surface electromyography (HD-sEMG) allows the extraction of local MU information and the decomposition of the HD-sEMG signals into the discharge times and motor unit action potentials (MUAPs) of individual MUs during isometric muscle contractions [8,9]. The properties of MUs that can be estimated from the single MUAPs and discharge times include the discharge rate (DR) of motor neurons, the DR variability, the force at which the MUs activate, known as the recruitment threshold (RT), and the velocity of propagation of the MUAPs across the muscle fibers, known as the motor unit conduction velocity (MUCV). These parameters provide direct information of neuromuscular function. For example, MU activity has recently been shown to be predictive of the maximum rate of force development [6], and the detrimental influence of aging on force stability has been associated with the variability of synaptic input common to motor neurons (for review, see [10]).

Despite these advances, HD-sEMG analysis still requires substantial expert intervention to ensure accurate parameter extraction and signal interpretation. Current gold-standard pipelines rely on semi-automatic processing stages that often require expert decisions, including spike-train editing, channel selection for MUCV estimation, and visual inspection during MU tracking [8,10]. These steps increase the analysis time and limit the scalability of HD-sEMG analysis in longitudinal and large-scale studies.

In this work, we present an automated pipeline for MU analysis from HD-sEMG signals acquired during isometric trapezoidal contractions. The main contribution of this study is the development and validation of an automated HD-sEMG analysis pipeline, with particular emphasis on stages that are commonly dependent on expert manual intervention, especially post-decomposition spike-train selection and validation (Section 2.3.2) and channel selection for MUCV estimation (Section 2.5.6). The proposed approach aims to reproduce expert-refined results while reducing operator dependency, thereby supporting more reproducible and scalable HD-sEMG analysis.

The implementation of the blind source separation (BSS)-based decomposition and automated spike-train selection procedures was evaluated using a public dataset containing signals from three muscles and the corresponding spike trains manually edited by multiple operators. The complete pipeline was then evaluated using HD-sEMG signals from the tibialis anterior (TA) muscle acquired from healthy volunteers in four sessions within one month.

## 2. Materials and Methods

The automated pipeline implemented in this study includes signal pre-processing, BSS-based decomposition, automated spike-train validation and selection, estimation of DR, RT, and MUAP waveforms, and automated channel selection for MUCV. In addition, within-session MU tracking was used to assess the repeatability of MU parameters across repeated contractions within each session.

**Figure 1:**
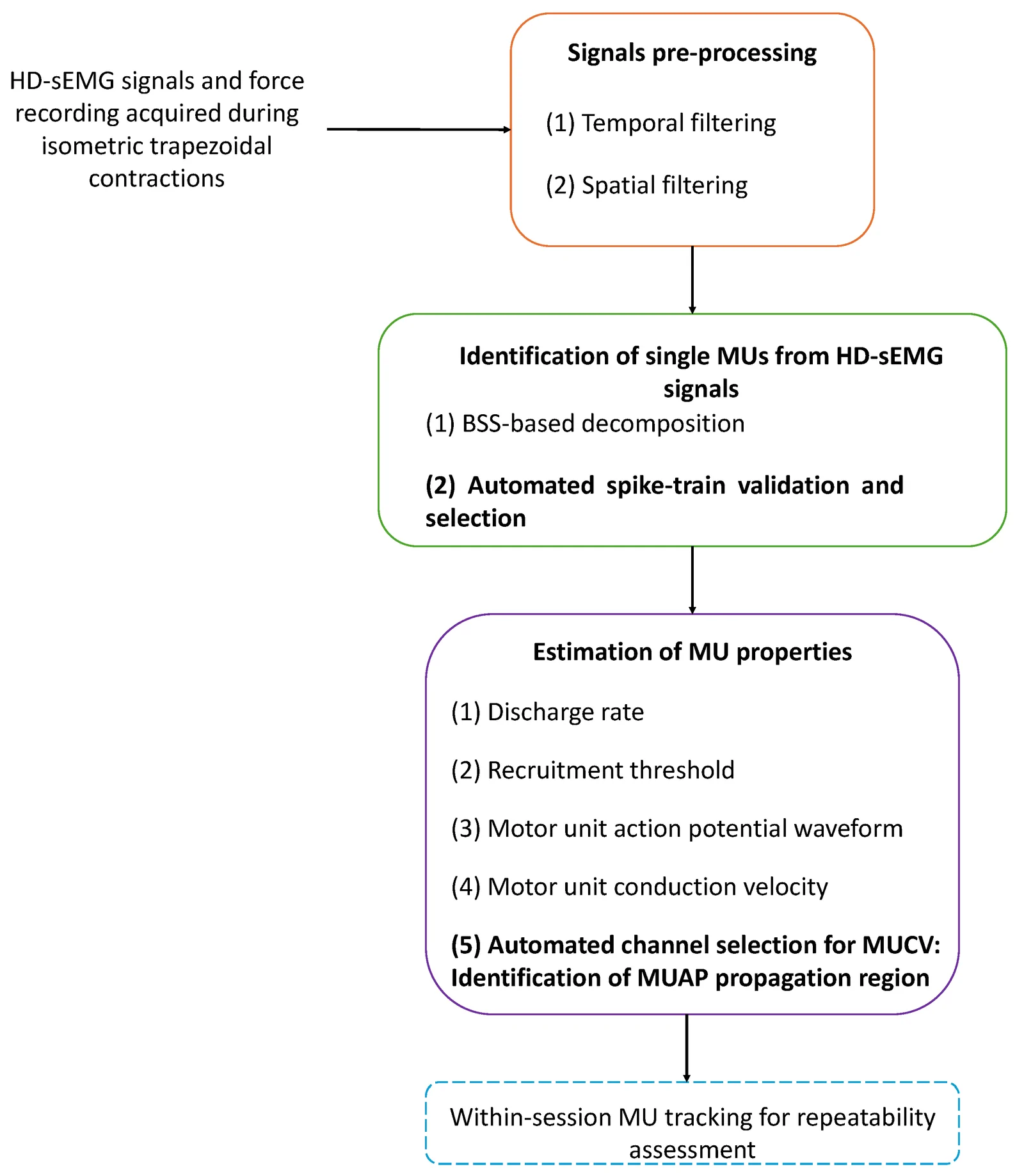
Automated pipeline for motor unit (MU) analysis from HD-sEMG signals acquired during isometric trapezoidal contractions. The pipeline includes pre-processing, BSS-based decomposition, automated spike-train validation and selection, and estimation of MU parameters, including automated channel selection for motor unit conduction velocity (MUCV) estimation.

Figure 1 summarizes the pipeline and highlights its main processing stages. The implementation details of each stage are described in the following sections. The pipeline was implemented in MATLAB R2023a using custom scripts.

### 2.1. HD-sEMG Acquisition During Isometric Trapezoidal Contractions

The accurate extraction and interpretation of MU parameters depend on appropriate electrode placement and the selected muscle contraction protocol. This section describes the conditions that HD-sEMG signals must satisfy to be analyzed with the proposed pipeline.

#### 2.1.1. Placement of the HD-sEMG Electrode Matrix

The innervation zone (IZ) refers to the muscle region where the neuromuscular junctions are concentrated. Identifying the IZ is important for HD-sEMG decomposition and estimation of MU parameters. Electrode placement relative to the IZ influences the number of identifiable MUs, as decomposition benefits from larger differences among MUAP waveforms, and it also affects the observation of MUAP propagation required for MUCV estimation. Previous studies have proposed placing the electrodes either between the IZ and the proximal or distal tendons of the muscle [4] or placing them centered above the IZ [10].

The HD-sEMG electrode matrix should be placed in a region that enables identification of the highest possible number of reliable MUs while also showing clear MUAP propagation across channels. Because this region may vary across muscles, candidate locations relative to the IZ should be evaluated during acquisition, and the position providing the best combination of MU identification and action potential propagation quality should be selected. An example of this procedure, applied to the TA muscle in the experimental evaluation in healthy volunteers, is presented in Appendix A.

#### 2.1.2. Measurement of Isometric Muscle Force

The isometric trapezoidal contraction protocol is commonly used to study MU recruitment strategies [4,8]. The proposed pipeline was developed for the analysis of HD-sEMG signals acquired during this contraction protocol, as it enables estimation of the RT and facilitates reliable HD-sEMG decomposition [10]. Therefore, the pipeline uses HD-sEMG signals acquired simultaneously with muscle force during isometric trapezoidal contractions. Each contraction consists of a force increase at a fixed rate, followed by a plateau phase at 20–80% of maximal voluntary force contraction (MVFC), and a final force decrease at the same rate.

### 2.2. HD-sEMG Signals Pre-Processing

#### 2.2.1. Frequency Filtering

HD-sEMG signals typically contain frequency components below 350 Hz [11]. Accordingly, a band-pass filter between 20 and 500 Hz (second-order zero-lag Butterworth) and a notch filter at 50 Hz (fourth-order zero-lag Butterworth) were implemented to remove low-frequency artifacts, preserve the relevant frequency band, and attenuate the power-line interference.

#### 2.2.2. Spatial Filtering

The longitudinal single differential (LSD) spatial filter is a technique used to attenuate common components such as artifacts, power line interference, and end-of-fiber effects from the monopolar HD-sEMG signals [1]. Hence, the LSD filter enables the identification of the IZ and the estimation of the MUCV [12,13]. The LSD was implemented by calculating the difference between the signals from adjacent channels vertically along the direction of the muscle fibers.

### 2.3. Identification of Single MUs from HD-sEMG Signals

#### 2.3.1. BSS Algorithm

The spatial and temporal information of the MUAPs is encoded in the HD-sEMG signals. However, direct interpretation of these signals is limited by several factors, including the properties of the biological tissues between the muscle fibers and the electrodes, the distance between them, and the muscle recruitment strategies (for review, see [14]). HD-sEMG decomposition allows identification of the electrical activity of individual MUs.

HD-sEMG signals can be modeled as a convolutive mixture of MU discharge times and MUAP waveforms. The discharge times can be estimated using BSS techniques [15]. The proposed pipeline integrates the BSS algorithm described by Negro et al. [15] to decompose the acquired HD-sEMG signals into candidate sources corresponding to the MU discharge times [15] and their corresponding spike-train representations. The spike-train representations consisted of binary trains, with ones at the identified discharge times and zeros elsewhere. In the present implementation, source estimation was performed over the entire signal recording using an extension factor of 1000/m [15], where m is the number of electrodes, and the source extraction procedure was repeated for 200 iterations to identify candidate sources.

#### 2.3.2. Automated Source-Validation and Spike-Train Selection

HD-sEMG signals are often decomposed only during the plateau phase of the contraction, and the final estimation of spike trains is then refined manually by an experienced operator [10,16]. During this manual editing stage, discharge times are removed or added based on the discharge-rate variability and unexpected discharge patterns identified by visual inspection. Following this manual editing, MU filters are recomputed to obtain the final estimate [10]. Despite its reliability, this procedure is subjective and time-consuming, often requiring more than 2 h to process a dataset of approximately 100 MUs [16].

To reduce the dependence on manual editing, we developed an automated source-validation and spike-train selection procedure to retain only reliable spike trains from the candidate sources identified using the BSS algorithm. In this automated procedure, the expected discharge patterns during the plateau phase of isometric trapezoidal contractions are used as the main criterion for source validation and spike-train selection. In these contractions, MU discharge rates increase after recruitment, stabilize during the force plateau, and decrease during force reduction. Therefore, low inter-spike interval variability is expected during the stable portion of the plateau [17,18,19]. This physiological behavior provides the basis for the proposed selection criteria.

The candidate sources and their corresponding spike trains are estimated over the entire recording. The stable portion of the plateau is then used as a decision window for automated source validation, duplicate identification and spike-train selection. Within this segment, spike trains with discharge rates exceeding 100 Hz are first excluded, as maximal discharge rates during slow isometric contractions typically range between 30 and 50 Hz (for review, see [20]). Spike trains containing fewer than five spikes during the plateau are also removed. Applying these exclusion criteria before duplicate identification prevents implausible spike trains from influencing the subsequent selection procedure.

Candidate spike trains corresponding to delayed or partial versions of the same MU are then identified using a discharge-time tolerance of ±2 samples. Two trains are considered duplicates when at least 50% of the discharges in the shorter train are matched to discharges in the other train. Among these duplicates, the train with the lowest coefficient of variation of inter-spike interval (CoV_ISI_) during the plateau phase is retained.

The plateau phase is identified from the simultaneously acquired force signal, and CoV_ISI_ is computed over the last 7 s of the plateau to avoid transient effects associated with force stabilization. This window was selected because the spike-train quality measures become more stable as the number of identified spikes increases [10] and because the plateau phase of trapezoidal contractions generally lasts longer than seven seconds. The selected duration should therefore be adapted according to the contraction protocol used. Finally, only spike trains with CoV_ISI_ values below 50% and pulse-to-noise ratio (PNR) > 28 dB are retained for subsequent analysis [15,21]. This PNR threshold has been associated with more than 85% correctly identified discharge times and a false alarm rate below 2% [22,23]. The plateau segment is used only to evaluate the exclusion, duplicate identification, and selection criteria. Once a spike train is retained, all of its discharges over the complete recording are preserved and used for estimation of MU physiological parameters. Throughout the procedure, exclusion and duplicate matching are performed using the spike-train representation, whereas PNR is evaluated using both the source and its corresponding spike train.

The default values of the selection parameters, including the plateau duration, minimum number of spikes, and upper discharge-rate limit, were defined using an independent dataset (SNCTP000003042-BASEC2017-01794). This dataset comprised three signals from the abductor pollicis brevis muscle, one signal from the gastrocnemius medialis (GM), and one signal from the biceps brachii muscle. Initial values were defined based on the physiological considerations described above and were then refined empirically through visual inspection of the resulting spike trains, with the objective of excluding implausible discharge patterns. During this refinement, the regularity of the inter-spike intervals and the sparseness of the discharge times were considered.

For the upper discharge-rate limit, values of 50 and 100 Hz were examined. A value of 100 Hz was retained as a physiologically plausible upper limit because MU discharge frequencies can reach 100–200 Hz during rapid contractions [24]. In addition, using a less restrictive threshold avoids the premature exclusion of spike trains with higher discharge rates and may facilitate future application of the algorithm in conditions in which discharge behavior differs from that observed in healthy volunteers. This filtering step was applied before duplicate identification because spike trains with implausibly high discharge rates could still show low CoV_ISI_ values and could therefore be incorrectly retained.

For the minimum number of spikes, candidate thresholds of 5 and 10 discharges were examined. A minimum of 10 discharges has previously been used as an exclusion criterion [25]. However, no relevant differences were observed in the final selected spike trains between these two thresholds. A threshold of five discharges was therefore retained because it is less restrictive and avoids discarding short but potentially valid spike trains before duplicate matching, while still excluding extremely short candidates with limited physiological plausibility.

The signals in the independent dataset did not include the recruitment and derecruitment phases. Therefore, the parameter-refinement procedure was performed only on the plateau phase, and the signals were segmented to include at least 10 s of isometric contraction [15] based on variations in signal amplitude.

Only one operator participated in this empirical refinement, and no blinding procedure was used at this stage.

The robustness of the final parameter values was subsequently evaluated through the sensitivity analysis described in Section 2.4.

### 2.4. Validation Against Manually Edited Spike Trains and Sensitivity Analysis

A public database [26] was used to validate the integration of the decomposition algorithm with the proposed automated source-validation and spike-train selection procedure. The algorithm parameter values used in this validation were those defined a priori. This database contains raw HD-sEMG signals recorded from the TA, gastrocnemius lateralis (GL), and GM muscles, along with MU spike trains manually edited by eight operators. The same dataset was also used to evaluate the robustness of the proposed selection procedure through a sensitivity analysis.

For validation, the raw signals from the public dataset were pre-processed using the band-pass filter (20–500 Hz) and 50 Hz notch filter described in the pre-processing stage (Section 2.2.1). The monopolar HD-sEMG signals were then decomposed using our implementation of the BSS-based algorithm, and the resulting candidate spike trains were processed using the proposed automated source-validation and spike-train selection procedure. The runtime of the complete automated procedure was measured across all signals in the dataset. Runtime measurements were obtained using MATLAB R2023a on Linux 5.16.18, with eight logical processing cores available to MATLAB.

The automatically selected spike trains were compared with the manually edited spike trains provided in the dataset using the rate of agreement (RoA), which ranges from 0 to 1, with 1 indicating identical spike trains [15]. A tolerance of ±2 samples was used to match discharge times. An automatically identified MU and a manually edited MU were considered a match when at least 50% of the discharges in the shorter train were matched in the other train. This criterion was used to avoid rejecting partial but plausible matches, given that, in specific cases, some operators included moderate-quality spikes whereas others did not, leading to only partial agreement even across manually edited spike trains [16]. Each automatically identified MU was matched to only one manually edited MU.

**Figure 2:**
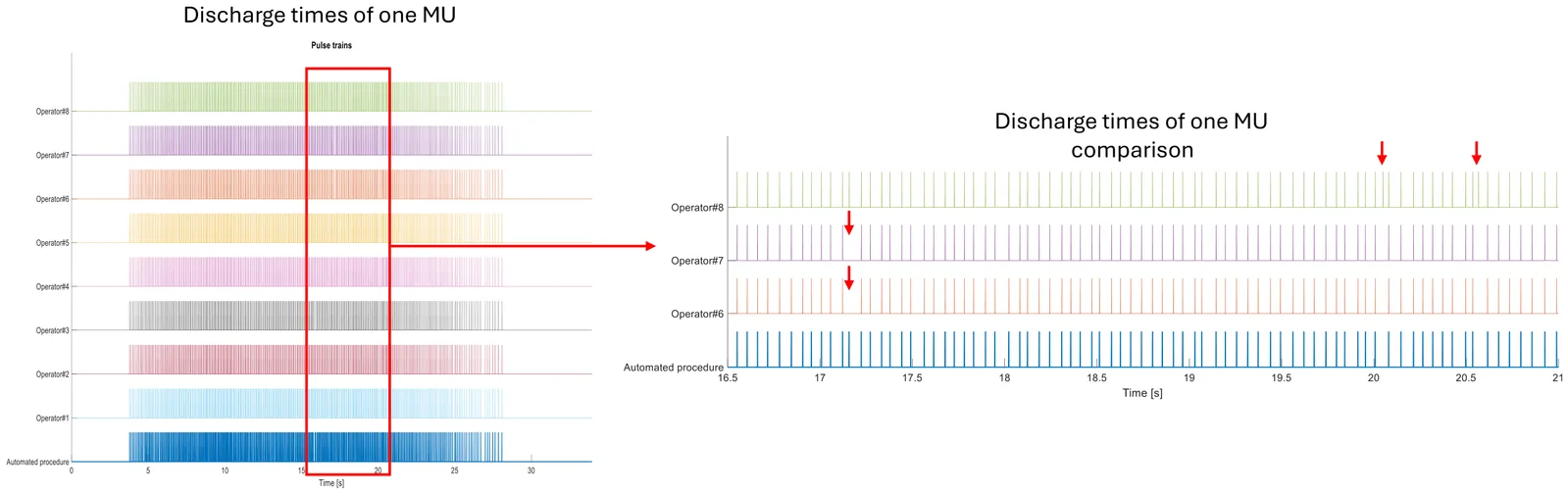
Representative comparison of discharge times for one matched MU between the proposed automated procedure and the spike trains manually edited by the eight operators. The figure shows a zoomed segment to highlight local differences in discharge-time identification.

RoA was computed separately against the spike trains edited by each of the eight operators. Figure 2 illustrates a representative comparison of the discharge times identified by the proposed procedure and by the eight operators for one matched MU, including a zoomed segment highlighting local differences in discharge-time identification. For each matched MU, agreement across operators was summarized using the median and interquartile range (IQR), and these per-MU values were then summarized for each signal. We verified that the same automatically identified MU was matched across operators whenever a matching reference MU was available. In cases where a MU was not matched because it had been excluded by some operators in the reference database, the IQR was computed using the available RoA values only.

As a complementary analysis, each operator was also compared with the remaining seven operators using the same matching procedure. For each MU, the RoA values obtained against the remaining operators were summarized by their median, allowing operator-wise agreement to be assessed using the same summary approach as for the proposed automated procedure.

Finally, two complementary analyses were conducted on the public dataset. First, a sensitivity analysis was performed to assess the robustness of the proposed selection procedure by varying the plateau duration (5, 7, and 10 s), the minimum number of spikes (3, 5, and 10), the upper discharge-rate limit (50, 75, and 100 Hz), and the CoV_ISI_ threshold (50, 70, and 100%). Second, the criteria for selecting among duplicate spike trains were compared: selection based on the lowest CoV_ISI_ during the plateau, the highest PNR, and the highest number of detected discharges.

For the sensitivity analysis, Friedman tests were used to evaluate differences in signal-level median RoA and matched MU fraction across parameter values. Signals were treated as repeated measures, with the tested values of each parameter considered as the factor levels. The Friedman test was selected because the same signals in the database were analyzed under all parameters setting, and both matched fraction and RoA could show skewed distributions. When significant effects were found, post hoc pairwise comparisons were performed with Bonferroni correction.

### 2.5. Estimation of MU Properties

**Figure 3:**
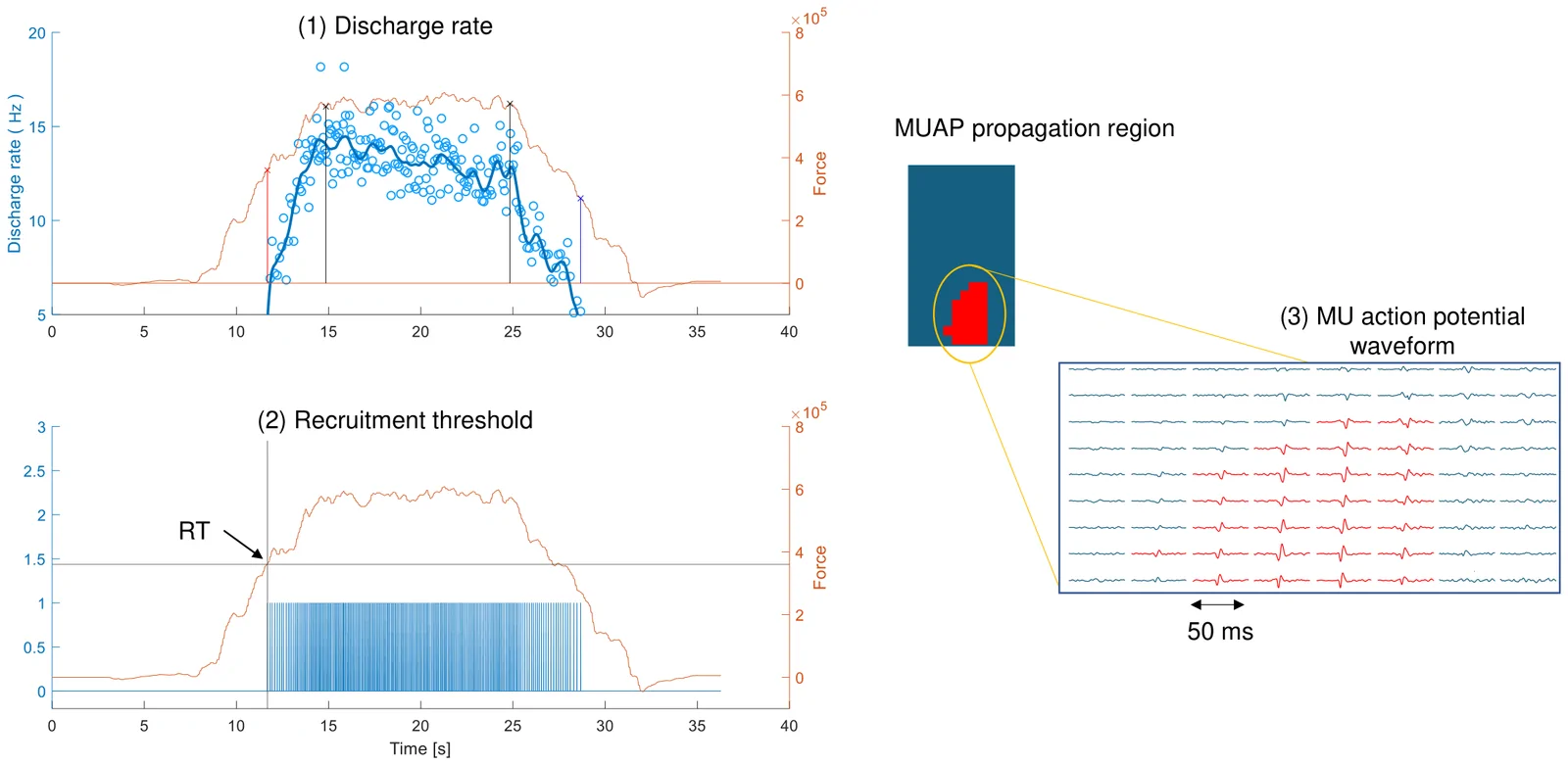
Physiological parameters estimated from identified MUs: (**1**) Mean discharge rate (DR) was calculated from a discharge-rate curve separately for the three phases of trapezoidal contraction. (**2**) Recruitment threshold (RT) corresponds to the muscle force at the first detected discharge. (**3**) Motor unit action potential (MUAP) waveforms were obtained by spike-triggered averaging over 50 ms windows after temporal and spatial filtering.

Several physiological properties can be estimated from the identified MUs to monitor the behavior of the neuromuscular system. In this study, the DR, RT, and MUCV were estimated for the identified MUs (see Figure 3).

#### 2.5.1. Discharge Rate (DR)

The DR was estimated from the binary spike trains of each MU, with ones at the identified discharge times. First, the binary spike train was low-pass filtered with a unit-area Hanning window of 1 s duration to obtain a discharge-rate curve across the contraction [27]. The mean DR was then computed separately for the ramp-up, plateau, and ramp-down phases of the trapezoidal contraction.

#### 2.5.2. Recruitment Threshold (RT)

The RT is defined as the force exerted by the muscle at the time of MU recruitment. For each MU, the RT was estimated as the force value at the occurrence of its first detected discharge [8].

#### 2.5.3. Motor Unit Action Potential (MUAP) Waveform

MUAP waveforms were estimated using the LSD-HD-sEMG signals. For each channel of the electrode matrix, the spike-triggered averaging was computed over 50 ms windows centered on the discharge times [28].

#### 2.5.4. Motor Unit Conduction Velocity (MUCV)

MUCV is defined as the velocity of propagation of the MUAPs along the muscle fibers. It was computed as the ratio between the inter-electrode distance (IED) and the time delay between MUAPs from adjacent channels within the same column.

**Figure 4:**
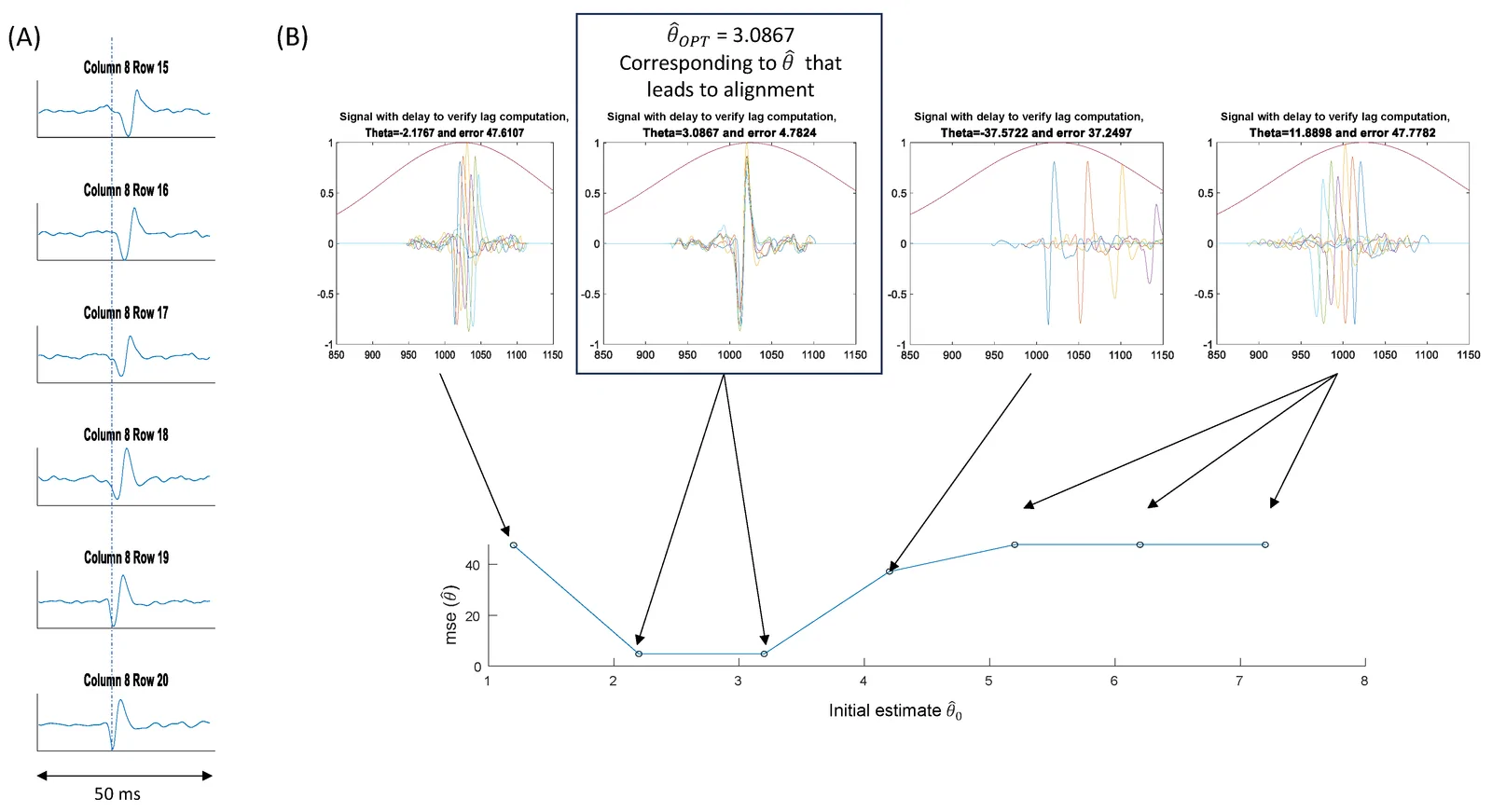
Grid search algorithm to estimate motor unit conduction velocity (MUCV) along muscle fibers: (**A**) MUAPs waveforms of the same MU extracted along six adjacent channels in the same column of the HD-sEMG matrix. (**B**) Newton’s method was initialized with delay values (${\hat{\theta }}_{0}$) ranging from 1.2 to 7.2. For each initial value, a converged delay estimate was obtained by minimizing the mse cost function. The final delay ${\hat{\theta }}_{OPT}$ was selected as the converged value with the minimum mse. In this example, the initial values ${\hat{\theta }}_{0}=2.2$ and ${\hat{\theta }}_{0}=3.2$ both converged to ${\hat{\theta }}_{OPT}=3.0867$, yielding a MUCV of 4 m/s. For visualization, the MUAP waveforms shown in (**A**) were shifted in time using each converged delay estimate to illustrate the resulting alignment, with a different color used to distinguish each channel.

The time delay was estimated using the maximum-likelihood algorithm [28], in which the Newton’s method is applied to minimize the mean square error (mse) cost function and identify the optimal delay ${\hat{\theta }}_{OPT}$ that best aligns the signals. Because convergence of the algorithm is sensitive to the initial delay value, a grid search procedure was implemented to initialize the Newton’s method. The initial time delay ${\hat{\theta }}_{0}$ was varied from 1.2 to 7.2 in steps of 1, with a tolerance of 0.2 (Figure 4). This range was selected because, for an IED of 4 mm and a sampling frequency of 3.07 kHz, the anatomical MUCV range corresponds to delay values between 1.9 and 6.14 samples [4]. The grid-search initialization reduced sensitivity to the starting value and provided final estimates of ${\hat{\theta }}_{OPT}$ within θ±0.02.

Only MUCV values within the physiological range (2–6.5 m/s) were retained [29].

#### 2.5.5. Verification of the MUCV Algorithm Implementation

The implementation of the MUCV algorithm, including the iterative initialization of the delay parameter ${\hat{\theta }}_{0}$, was verified against the OTBiolab software [30], which implements the reference algorithm described in [31] using a Gaussian window. For this comparison, four channels were selected from a HD-sEMG signal of the abductor digiti minimi acquired with a 64-electrode grid (8 mm IED, sampling frequency 2048 Hz) and provided by the software.

The delay between channels was estimated using both implementations. Because the conduction velocity was estimated directly from the signals without decomposition, the resulting value corresponded to the average propagation velocity of action potentials along the muscle fibers, i.e., the muscle fiber conduction velocity (MFCV).

The agreement between the two implementations was quantified using the relative error of the estimated MFCV. The MFCV was estimated from a selected 50 ms signal segment across the four channels. The values obtained with OTBiolab software and our implementation were 4.33 m/s and 4.34 m/s, respectively, corresponding to a relative error of 0.3%.

#### 2.5.6. Automatic Channel Selection for MUCV

Reliable estimation of the MUCV requires the selection of adjacent channels along the muscle-fiber direction that contain the same propagating MUAP while excluding noisy channels.

**Figure 5:**
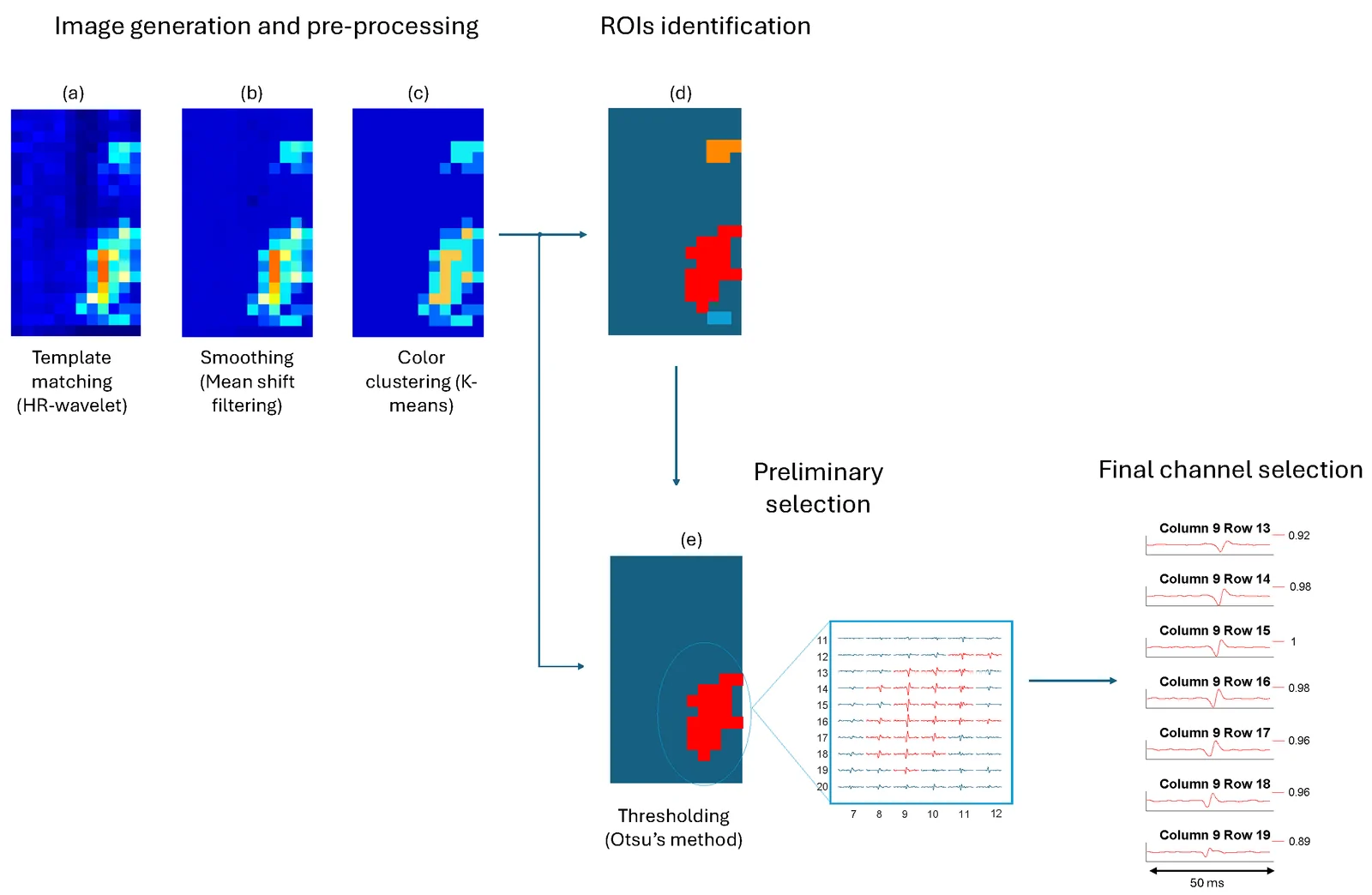
Automatic channel selection for MUCV estimation. The algorithm identifies the MUAP propagation region and selects the final channel set for MUCV estimation. (**a**) A template-matching image was generated using the first-order Hermite–Rodriguez (HR) wavelet. (**b**,**c**) Mean-shift filtering and k-means clustering (k = 4) were applied to smooth and simplify the image. (**d**) A binary image was created by thresholding the cluster levels, and regions of interests (ROIs) were identified using connected-components analysis. (**e**) Otsu’s thresholding was then applied to refine ROI selection. Candidate columns were evaluated using a cross-correlation threshold greater than 0.8. The algorithm first searched for columns containing at least five adjacent channels and fell back to four channels only when no valid five-channel column was available.

We implemented an image-processing approach that treats each electrode as a pixel to automatically identify the MUAP propagation region and select the most suitable column for MUCV estimation. By using template matching to analyze the spatial similarity of MUAPs, the proposed method reduces the dependence on manual visual inspection, in contrast to previous approaches in which the channel selection was based on operator inspection or waveform similarity across channels within the same column [4,8]. Figure 5 presents a graphical overview of the algorithm. First, a template-matching image was generated using the first order Hermite–Rodriguez wavelet, which has a shape similar to MUAP waveforms in single differential recordings [32]. This step allows the identification of electrodes containing action potentials. The resulting image was then smoothed using mean-shift filtering, followed by k-means clustering with k = 4 to highlight channels with similar waveform characteristics. The four clusters were subsequently binarized by assigning the two lower-intensity clusters to zero and the two higher-intensity clusters to one. Regions of interests (ROIs) were then identified in the binarized image using the 4-connected adjacency criterion.

When only one ROI was identified, the corresponding channels were considered the MUAP propagation region. When multiple ROIs were identified, an additional threshold calculated using the Otsu’s method was applied [33]. Otsu’s method was used because it automatically determines the optimal threshold by maximizing separation between intensity classes.

Within the detected MUAP propagation region, candidate columns were evaluated using a cross-correlation threshold greater than 0.8 [4]. Within each column, only the group of adjacent channels fulfilling this criterion was retained. For MUCV estimation, the algorithm first searched for columns containing at least five adjacent channels and then fell back to four channels only when no valid five-channel column was available. When more than one column satisfied this criterion, the final column was selected using a ranking procedure based on the mean cross-correlation and the template-matching coefficients within the detected MUAP propagation region.

This automatic channel-selection algorithm was evaluated using two complementary approaches, detailed in Section 3.6.

## 3. Experimental Evaluation in Healthy Volunteers

To evaluate the proposed pipeline, an experimental study was conducted using HD-sEMG signals acquired from the TA muscle during isometric trapezoidal contractions performed at different force levels. The simultaneous acquisition of HD-sEMG signals and force enabled the estimation of the corresponding MU physiological parameters and supported the evaluation of their physiological plausibility, as well as their within-session repeatability.

### 3.1. Participants

Nine healthy volunteers (mean age: 27 ± 6 years old; range: 19–41 years; 2 women, 7 men) were enrolled in this study. All participants agreed to participate in the clinical investigation and provided written informed consent before participation. The study (SNCTP000005276|BASEC2022-D0105) was approved by the Ticino’s cantonal ethics committee and Swissmedic and was conducted in accordance with the Declaration of Helsinki. All volunteers were healthy adults with no significant disease condition or skin disorders/allergies at the site of contact with the HD-sEMG device. Participants completed the Global Physical Activity Questionnaire [34], and all met the recommendations on physical activity for health. All volunteers reported the right leg as their dominant leg.

### 3.2. Experimental Procedures

The experimental procedures were executed in four sessions: session 1, after 1 h (session 2), after 15 days (session 3), and after 30 days (session 4) from session 1. In each session, HD-sEMG and force signals were acquired simultaneously from the TA muscle of the dominant leg.

The volunteers performed the experimental procedures while seated on a fowler bed, with the hip flexed at 38–40° and the leg extended. The leg was positioned over the force measurement device with the ankle at 30°. Straps were used to restrict movement and ensure isometric contractions. Before electrode placement, the skin was cleaned with abrasive gel and alcohol to reduce the electrode–skin impedance [11]. Then, the electrode–skin interface was filled with a conductive cream (Spes Medica, Genova, Italy). Prior to the experiment, the volunteers performed a trial of the isometric trapezoidal contractions.

In each session, the volunteers first performed three MVFCs separated by 30 s. They were instructed to “push as hard as possible” and hold for at least 3 s [4]. The highest force value was used to define the target levels for the subsequent trapezoidal contractions.

The trapezoidal contractions were then performed at three target force levels corresponding to 35%, 50% and 70% of MVFC, representing low, moderate, and high levels of muscle activation. The volunteers were instructed to follow a force profile displayed on a screen, in which they had to increase the force at a rate of 5% MVFC/s until reaching the target force, maintain for 10 s, and then decrease the force to rest at the same rate. The volunteers received visual feedback of the force exerted by the muscle through the task. The trapezoidal contractions were organized into two series of three contractions, with one contraction at each force level per series. Contractions were separated by 3.5 min.

In order to assess repeatability, an additional series was executed by 4 of the 9 volunteers after removing the HD-sEMG electrode matrix, cleaning the skin, and repositioning the matrix.

### 3.3. HD-sEMG and Force Acquisition

**Figure 6:**
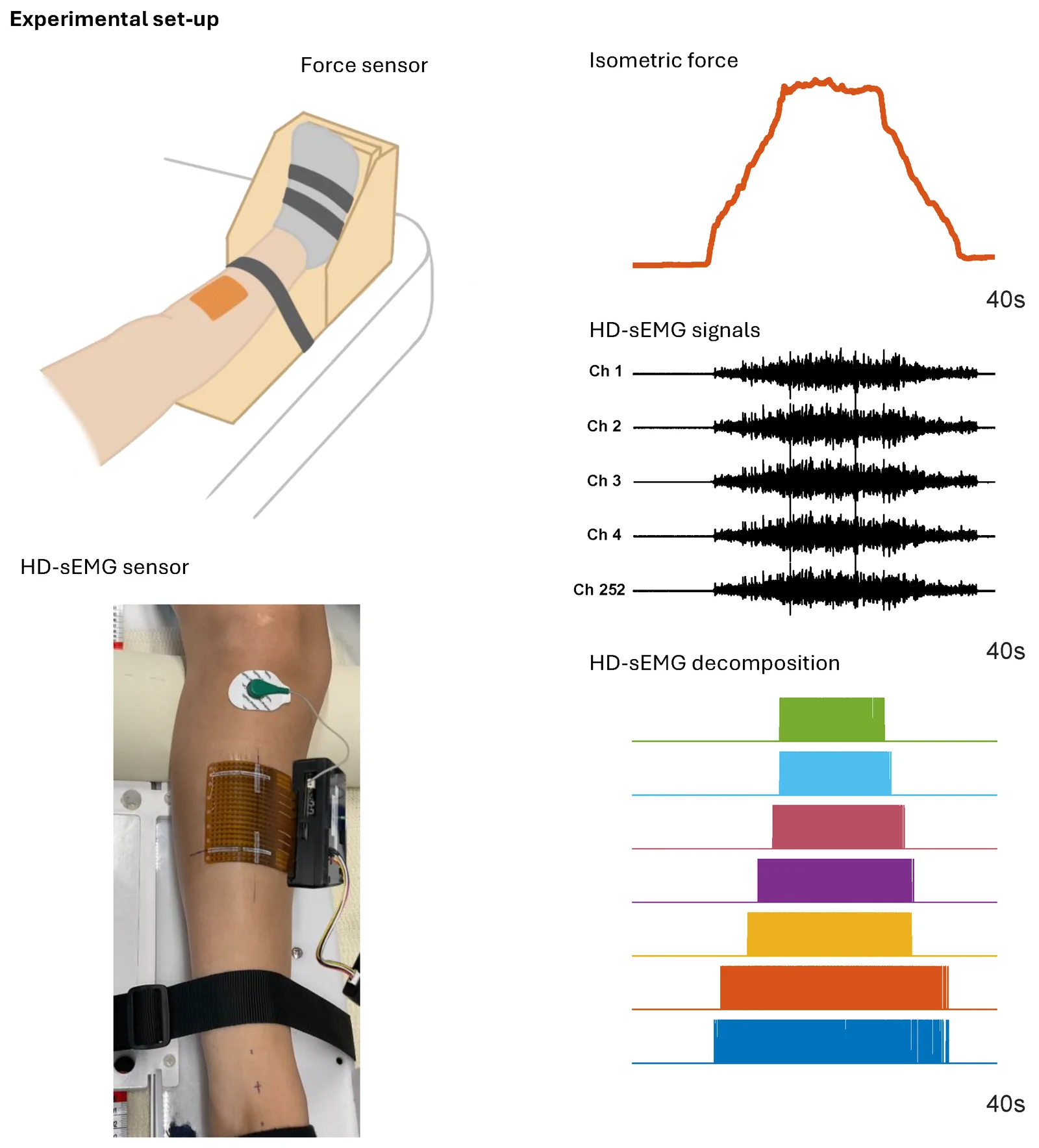
Experimental setup for simultaneous acquisition of HD-sEMG signals and ankle dorsiflexion force during isometric trapezoidal contractions using the High-Density Modular surface electromyography (HD-M-sEMG) prototype developed at our institute. An example of one trapezoidal contraction performed by a volunteer is shown, including the measured isometric ankle dorsiflexion force, HD-sEMG signals from five channels, and the spike trains of seven MUs identified from the HD-sEMG signals, with each MU represented by a different color.

The experimental setup for simultaneous acquisition of HD-sEMG signals and ankle dorsiflexion force during isometric contractions of the TA muscle is illustrated in Figure 6.

A custom device was developed to measure isometric ankle dorsiflexion force simultaneously with the HD-sEMG acquisition. The device secured the leg in a comfortable and reproducible position across contractions. Force was measured using a 10 kg load cell (SparkFun Electronics, Niwot, CO, USA) positioned parallel to the foot base. The signal from the load cell was amplified with a gain of 128 using an HX711 (Avia Semiconductor, Xiamen, China), acquired at 10.5 Hz using an STM32F103C8 (STMicroelectronics, Geneva, Switzerland), and transmitted to the computer. The force-measurement device was connected to a graphical user interface, which provided visual feedback of the muscle force. The force trajectories were stored both by the HD-sEMG device and by the graphical user interface to enable data synchronization and subsequent analysis.

HD-sEMG signals were acquired using the HD-M-sEMG, a modular prototype developed by the Institute of Systems and Applied Electronics at University of Applied Sciences and Arts of Southern Switzerland (SUPSI). The device is composed of four units that enable the simultaneous acquisition, processing, and storage of 252 sEMG signals. Signal detection was performed using a disposable rigid–flexible electrode array with 252 silver-coated contacts (12 columns × 21 rows; diameter 1.95 mm; IED 4 mm). Signal conditioning and digitization were performed by four RHD2164 digital electrophysiology interface chips (Intan Technologies, Los Angeles, CA, USA), and digital processing and data storage were handled by an SMF2000 FPGA module (Trenz Electronic GmbH, Hüllhorst, Germany).

Table 1 presents a comparison between the front-end specifications and electrode-array geometry of the implemented system with the reference values reported in the literature [11]. Although the amplifier common mode rejection ratio (CMRR) at 50 Hz (82 dB) was below the theoretical reference value, the battery-powered configuration and skin treatment reduced common-mode interference. All other system specifications were within the recommended ranges [11].

#### Placement of the HD-sEMG Electrode Matrix

To identify the optimal region for HD-sEMG acquisition from the TA muscle and enable accurate estimation of MU parameters, three electrode-matrix locations relative to the IZ were compared in five healthy volunteers: above, below, and centered over the IZ. The results of this preliminary study indicated that the optimal placement was the region above the IZ (Appendix A).

**Figure 7:**
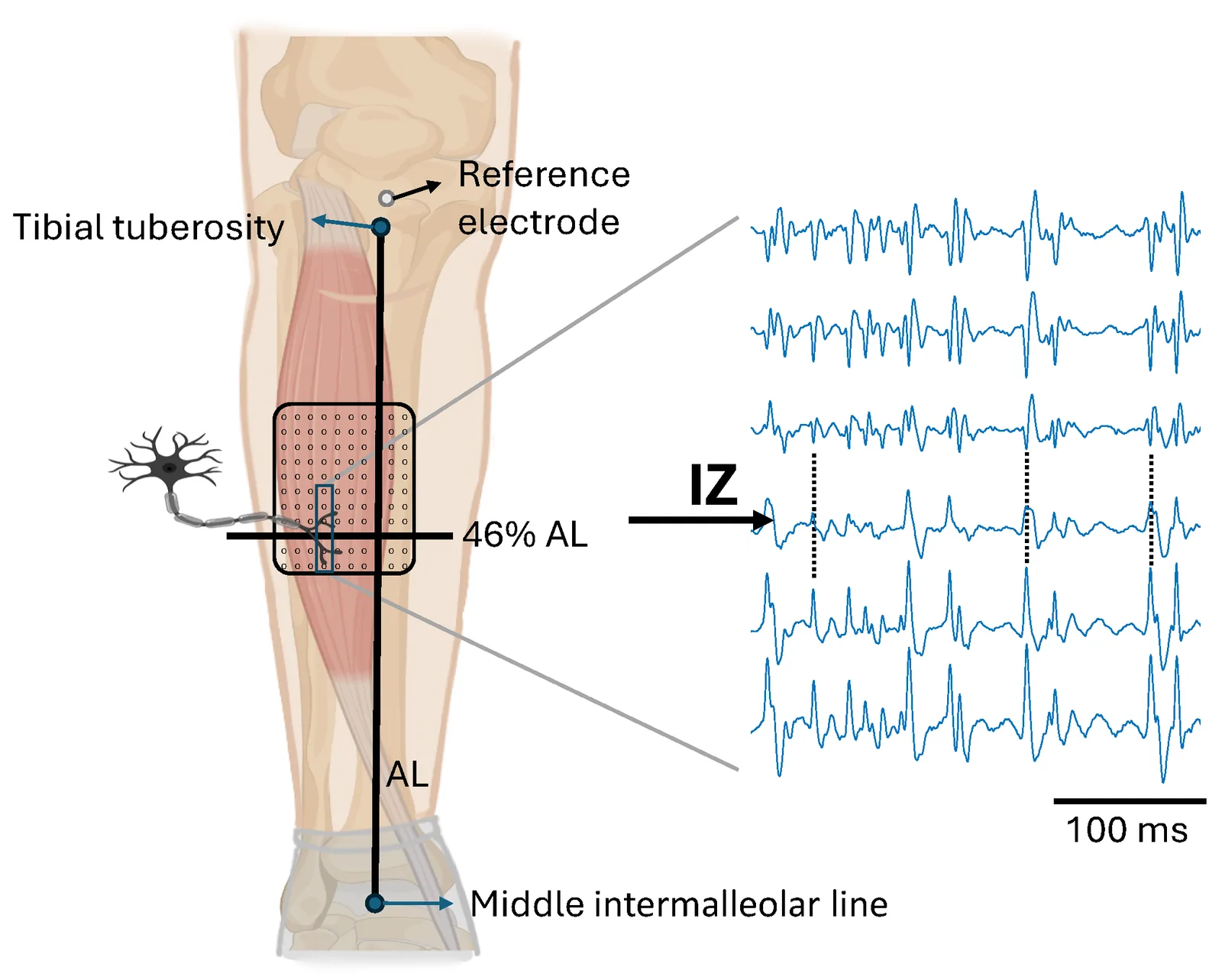
Anatomical lines used to standardize electrode placement over the TA muscle. The optimal region was identified above the innervation zone (IZ). The IZ can be identified in spatial filtered HD-sEMG by low amplitude and phase inversion and was typically located at 46% of the anatomical landmark (AL) measured from the tibial tuberosity to the intermalleolar line. Figure created with BioRender.

To standardize the electrode placement based on these results, two anatomical reference lines were marked on the skin (Figure 7). First, the anatomical landmark was drawn: a longitudinal line between the tibial tuberosity and the intermalleolar line [1]. Second, a transverse line was drawn at 46% of the length of this longitudinal line measured from the tibial tuberosity, with the ankle positioned at 90°, corresponding to the median IZ location identified in the preliminary study. The electrode matrix was then positioned proximally, parallel to the tibia and therefore to the direction of MUAP propagation, with the third column over the longitudinal line and the third row, from bottom to top, over the transverse line. The reference electrode was placed over the tibial tuberosity.

### 3.4. Estimation of Motor Unit Parameters

The acquired HD-sEMG signals were filtered in time and space as described in Section 2.2. After filtering, the signals were decomposed using our implementation of the decomposition algorithm together with the automated spike-train selection and validation procedure described in Section 2.3. Before parameter estimation, an additional outlier-exclusion step was applied: spikes corresponding to negative peaks in the sources were removed from the spike trains, and spikes associated with instantaneous discharge rates above 60 Hz were excluded. This threshold was chosen because discharge rates during high-force isometric contractions in healthy volunteers (>80% MVFC) are typically reported below or around 60 Hz (for review, see [35,36]); since the contractions analyzed in our experimental protocol were performed at lower force levels, higher values were classified as potential outliers. The PNR was then evaluated, and only spike trains with PNR >28 dB were retained. Spike trains with fewer than 30 discharges were also excluded because previous studies have reported that approximately 30–100 spikes are generally sufficient for the reliable extraction of MUAP waveforms [10], which was required for MUCV estimation and tracking. In addition, spike trains containing inter-spike intervals (ISIs) longer than 2 s were excluded to ensure reliable RT estimation [5]. Finally, sources that remained active throughout the recording, including the rest period, were excluded because they were considered more likely to reflect power line interference than physiological activity. All these steps were performed automatically.

Once the discharge times of the individual MUs had been identified, the DR, RT, and MUCV were estimated for each of the isometric trapezoidal contraction performed in all four sessions.

### 3.5. Statistical Analysis

The descriptive statistics of the estimated parameters were then calculated to assess whether the obtained values were within previously reported physiological ranges. In addition, the number of detected MUs was quantified for each contraction to evaluate the consistency across contractions and sessions.

To assess whether the estimated MU parameters also reproduced expected force-dependent patterns, a linear mixed-effects model was fitted separately for each parameter using R version 4.4.2 (lmerTest). These analyses were designed to compare the parameters of MU populations identified across target-force levels rather than to assess changes in individual MU behavior across contractions, as MUs were not tracked across contractions performed at different force levels. The target force was defined as the fixed effect, with random intercepts for volunteer, session nested within volunteer, and contraction. Each contraction was represented by a unique identifier. The Kenward–Roger method was used to approximate the degrees of freedom and obtain the corresponding *p*-values.

In a separate analysis, to assess the variability associated with electrode placement and the within-session repeatability of the estimated parameters, MUs identified before and after electrode removal and repositioning were tracked independently within each session. For this purpose, MUAP waveforms from different contractions were compared using the normalized 2D cross-correlation [5]. Tracking was performed between the contractions in the two series acquired before repositioning and the additional series acquired after repositioning the electrode matrix. For each force level, only the contraction pair with the highest number of tracked MUs was retained. Finally, the physiological parameters of the tracked MUs were compared using the Pearson correlation.

### 3.6. Automatic Channel-Selection Validation

To assess the consistency of the automatic channel-selection algorithm for MUCV estimation, a complementary analysis was performed on the tracked MUs before and after electrode repositioning. In a first condition, MUCV was computed using the same set of electrode channels in both contractions, selected from the two automatically identified channel sets as the one yielding the lower mse after waveform alignment. In a second condition, MUCV was computed using the channel set selected independently by the algorithm in each recording. Pearson correlation was then used to compare the resulting MUCV estimates before and after electrode repositioning.

In addition to this consistency analysis, the automatic channel-selection algorithm was compared with manual channel selection in a representative subset of MUs. Only Session 3 was considered, and the subset was restricted to MUs for which the MUCV algorithm successfully converged and produced an estimate within the physiological range of 2–6.5 m/s, as described in Section 2.5.4. For each volunteer and target-force level, the contraction containing the largest number of eligible MUs was selected. Up to three MUs were then randomly selected from each contraction using a fixed random seed. For one volunteer, only two eligible MUs were available at each target-force level, resulting in a total of 78 MUs for visual inspection and manual channel selection.

A custom graphical user interface was implemented to display the longitudinal single differential MUAP waveforms across 20 rows and 12 columns of the electrode grid and to enable manual channel selection. The MUs included in the manual assessment were presented in randomized order, and neither the automatically selected channels nor the corresponding MUCV estimates were displayed during manual selection.

Manual channel selection was performed by identifying an electrode column showing MUAP propagation and selecting a contiguous set of at least four adjacent channels with minimal variation in waveform shape. Selections containing fewer than four channels, nonadjacent channels, or channels from different columns were rejected, and the operator was prompted to perform a new selection. After manual selection, MUCV was estimated using the selected channels, and the cross-correlation between adjacent channels was calculated to quantify the waveform-shape similarity across the selected channels. The runtime of the complete manual-analysis procedure was measured, including the time required to calculate the adjacent-channel cross-correlation and estimate MUCV.

The automatically and manually selected channel sets were compared using the Euclidean distance between their central positions, and the absolute differences in row and column position. Agreement between the MUCV estimates obtained using the automatically and manually selected channel sets was assessed using Pearson correlation. First, the analysis included all MUs for which the manually selected channel set produced a numerical MUCV estimate. The correlation analysis was then repeated after excluding MUCV estimates obtained from manually selected channel sets that fell outside the physiological range of 2–6.5 m/s.

## 4. Results

### 4.1. Validation on the Public Dataset

The proposed decomposition and automated spike-train selection procedure was first evaluated on the public database of HD-sEMG signals with manually edited spike trains. Validation was performed using the default parameter values defined in Section 2.3, and the RoA was used to quantify agreement between the automatically selected spike trains and the manually edited spike trains. The results are presented below, including the number of matched MUs, agreement across operators, sensitivity to the main selection parameters, and comparison of duplicate selection criteria.

#### 4.1.1. Number of Identified MUs

Table 2 summarizes the number of MU spike trains identified by the proposed automated procedure and compares these values with those provided in the database using the semi-automatic approach [16]. The table also shows that the number of manually retained MUs varied across operators for the same signal, as indicated by the min–max range of manual MUs. Across the dataset, the automatic procedure matched 106 of the 135 MU spike trains available in the database (78.5%), while 30 reference MUs were not identified. In addition, the automatic procedure detected 47 extra MUs, resulting in a higher number of identified MUs for most signals.

#### 4.1.2. Agreement with Manually Edited Spike Trains Across Operators

Table 3 presents the agreement between the automatically selected spike trains and the manually edited spike trains. For each matched MU, the RoA values across operators were summarized using the median and IQR. These MU-level values were then summarized across MUs, again using the median and IQR, to obtain one set of agreement values per signal. The median of the MU-level median RoA was consistently high across signals, with most values between 0.96 and 0.99. In addition, the IQR of the MU-level median RoA was low for most signals, indicating consistent agreement across matched MUs within each signal. Variability across operators was also small, as represented by the low median and IQR of the MU-level operator IQR values.

Overall, these results indicate that the proposed automated procedure reproduces manually edited spike trains with high agreement.

**Figure 8:**
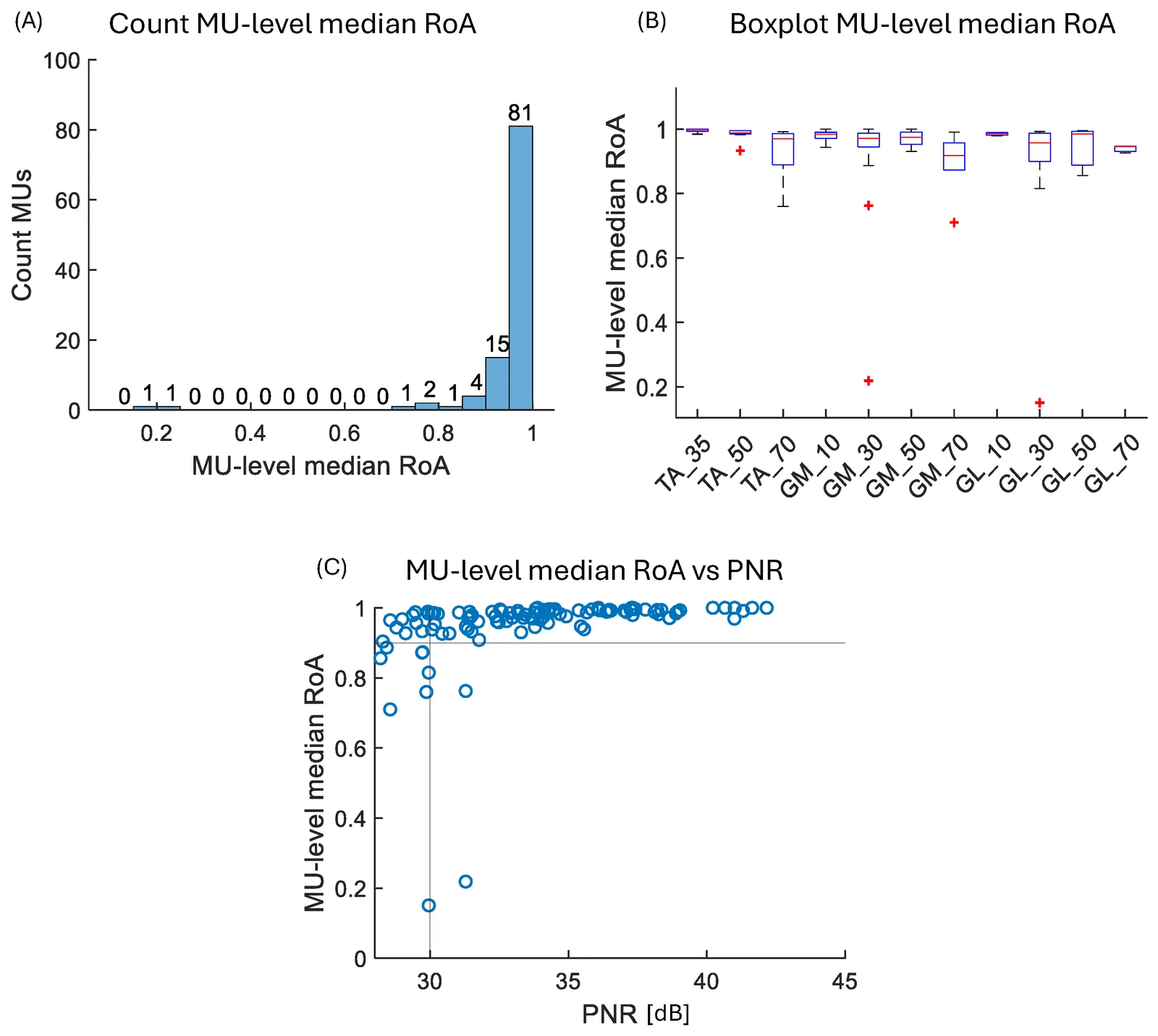
Descriptive analyses of agreement between our proposed automated procedure and the manually edited spike trains. (**A**) Count MU-level median RoA: Distribution of MU-level median RoA values across the 106 matched MUs; the overall median was 0.98. (**B**) Boxplot of MU-level RoA values for each signal, presenting possible outliers. Red crosses denote outliers. (**C**) MU-level median RoA vs. PNR: Scatter plot of PNR versus MU-level median RoA, showing that lower-agreement cases were mainly concentrated between the 28 dB acceptance threshold and 30 dB. The vertical and horizontal lines indicate PNR = 30 dB and MU-level median RoA = 0.9, respectively.

Figure 8 presents three descriptive analyses of agreement with the manually edited spike trains: the distribution of MU-level median RoA values (Figure 8A), a boxplot of these values for each signal (Figure 8B), and a scatter plot of median RoA as a function of PNR (Figure 8C). Among the 106 matched MUs, 96 showed a median RoA above 0.9 (90.6%). Only a small number of matched MUs showed lower agreement values (8/106), with three MUs between 0.7 and 0.8, and five MUs between 0.8 and 0.9 (Figure 8A). The overall median was 0.98. This result is consistent with the public database itself, in which inter-operator agreement was reported to be very high overall (median RoA = 0.996) [16], although a small number of MU-level median RoA values were also below 0.9 when each operator was compared with the remaining seven. In this complementary operator-wise analysis, 5 of 113 MU-level median values were below 0.9, including 3 for operator 2, 1 for operator 4, and 1 for operator 8, with one value at 0.6. In the database study, low inter-operator agreement values were attributed in specific cases to failure to recalculate the MU filter over the whole contraction or to inclusion of moderate-quality spikes by some operators [16].

Figure 8B also shows two MUs with RoA values around 0.2 for two signals acquired from the GM at 30% MVFC and from the GL at 30% MVFC. In both cases, at least 50% of the spike trains from the operators were contained in the spike train identified by the automated algorithm, suggesting that these cases correspond to partial but plausible matches and may be considered outliers relative to the general distribution of the agreement values.

To explore the effect of the PNR threshold on agreement, a scatter plot of PNR versus RoA was also generated (Figure 8C). Lower-agreement cases were mainly concentrated near the acceptance threshold, between 28 and 30 dB. Among the total matched MUs, 19 had PNR values below 30 dB, and 11 of these still showed RoA values above 0.9. Taken together, the scatter plot (Figure 8C) and these counts suggest that reduced decomposition quality may contribute to some of the observed discrepancies. Nevertheless, high agreement was still achieved for several MUs with PNR values close to the acceptance threshold and for almost all MUs above it, with only two exceptions.

#### 4.1.3. Sensitivity Analysis

**Figure 9:**
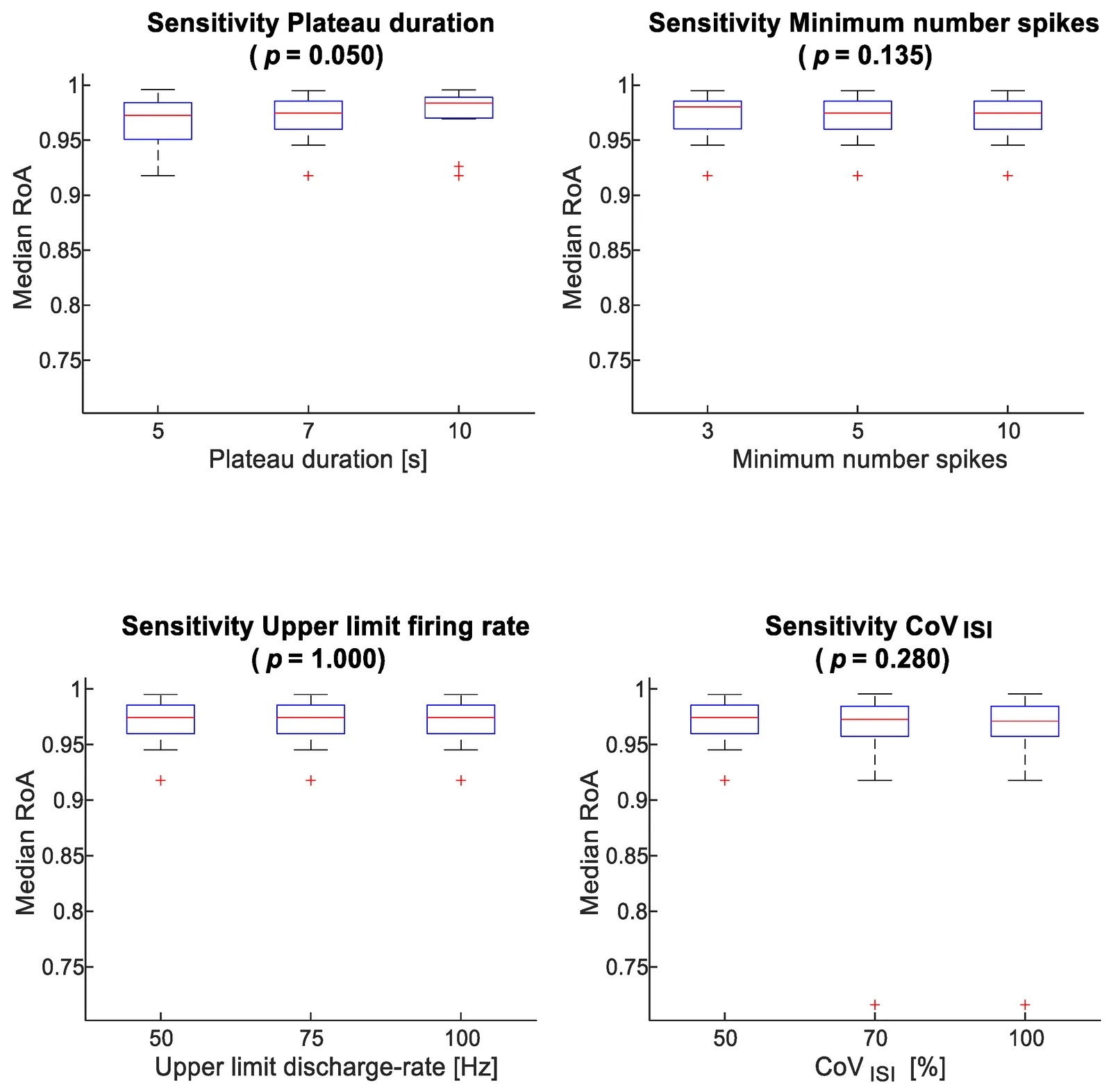
Sensitivity analysis of the proposed spike-train selection procedure with respect to the signal-level median RoA. Boxplots show the distribution across the 11 analyzed signals obtained by varying, one factor at a time, the plateau duration (5, 7, and 10 s), the minimum number of spikes (3, 5, and 10), the upper discharge-rate limit (50, 75 and 100 Hz), and the CoV_ISI_ threshold (50, 70, and 100%), while keeping the remaining parameters fixed at their default values.

**Figure 10:**
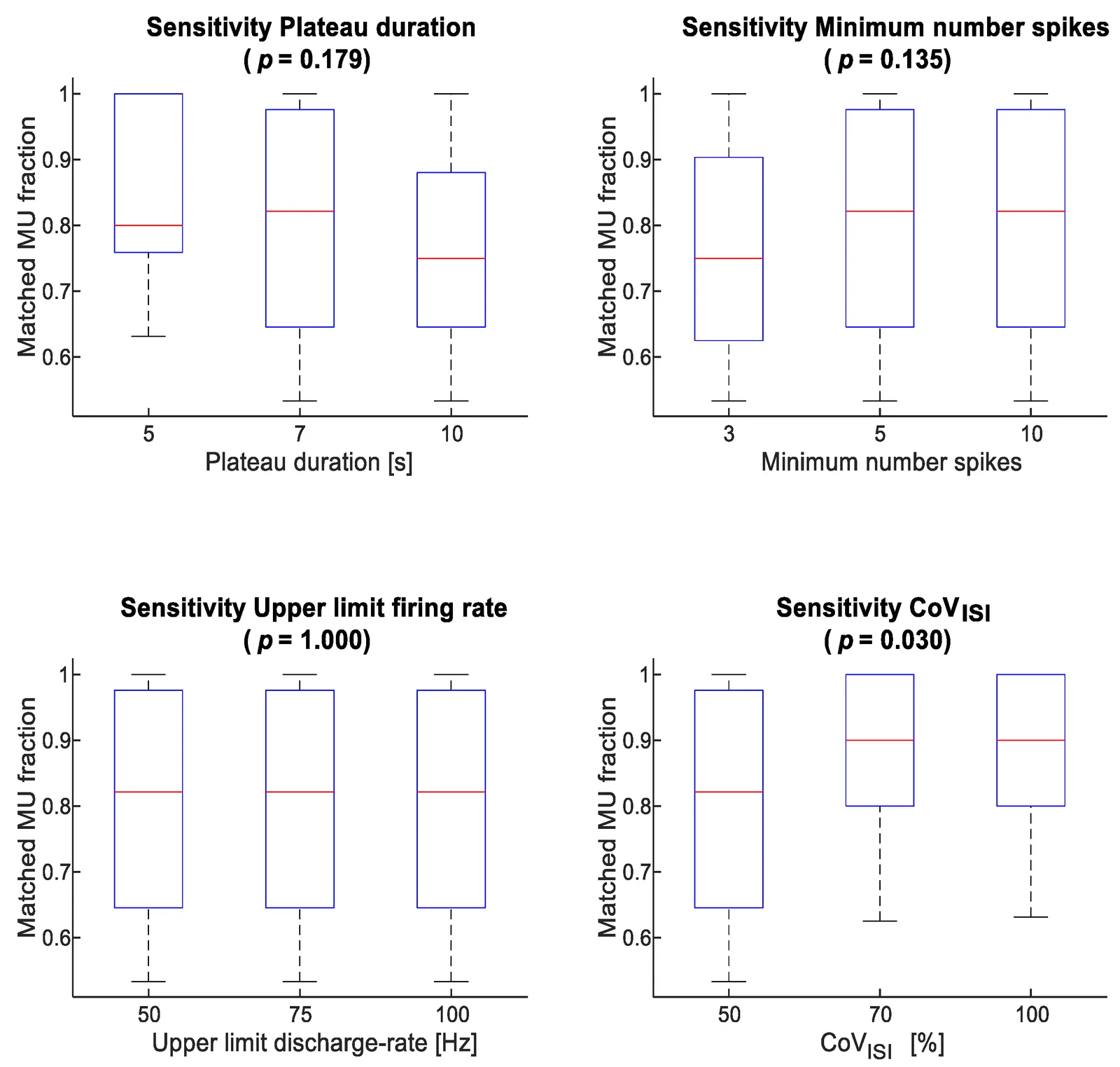
Sensitivity analysis of the proposed spike-train selection procedure with respect to the matched MU fraction. Boxplots show the distribution across the 11 analyzed signals of the fraction of matched MU spike trains between the public database and the proposed algorithm when varying the parameters one factor at a time.

To assess the robustness of the proposed spike-train selection procedure, a sensitivity analysis of the main parameters was performed. Figure 9 and Figure 10 summarize the results obtained by varying the tested parameter values: plateau duration (5, 7, and 10 s), minimum number of spikes (3, 5, and 10), upper discharge-rate limit (50, 75, and 100 Hz), and CoV_ISI_ threshold (50, 70, and 100%). Friedman tests were used to evaluate differences in signal-level median RoA and matched MU fraction across parameter values.

The plateau duration significantly affected the signal-level median RoA ($\chi^{2}=6$, *p* = 0.0498). Post hoc analysis showed a significant difference between 5 s and 10 s (*p* = 0.0439), with higher agreement for the longer duration. This is consistent with the use of a longer stable plateau segment for CoV_ISI_ estimation, which provides more stable spike train quality measures. In contrast, the effect of the plateau duration on the matched MU fraction was not significant ($\chi^{2}=3.44$, *p* = 0.179).

The CoV_ISI_ threshold did not significantly affect the signal-level median RoA ($\chi^{2}=2.54$, *p* = 0.28), but it significantly affected the matched MU fraction ($\chi^{2}=7$, *p* = 0.0302). Post hoc analysis showed a significant difference between CoV_ISI_ thresholds of 50 and 100 (*p* = 0.037). Although the CoV_ISI_ threshold did not significantly affect the signal-level median RoA, lower RoA values were observed for the more permissive thresholds (70%, and 100%). In particular, the minimum median RoA was 0.72 for CoV_ISI_ thresholds of 70 and 100, whereas it was 0.92 for CoV_ISI_ = 50. Overall, these results indicate that the CoV_ISI_ threshold is a key parameter that primarily controls the trade off between MU matching and the inclusion of MUs with lower agreement.

Neither the minimum number of spikes nor the upper discharge-rate limit showed a significant effect on the signal-level median RoA or matched MU fraction (*p* > 0.05).

#### 4.1.4. Comparison of Duplicate Selection Criteria

**Figure 11:**
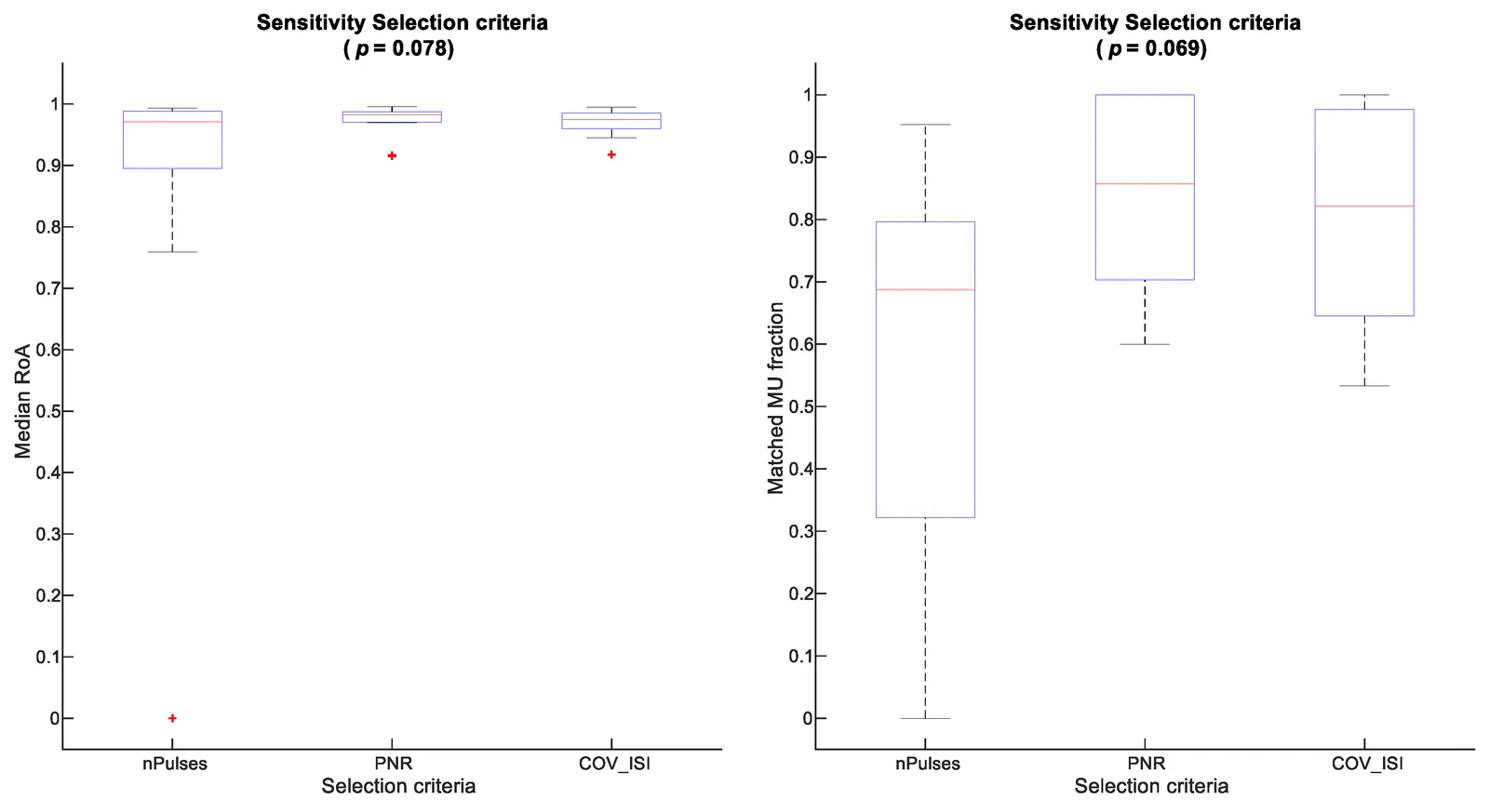
Comparison of the criteria used to select among duplicate spike trains: selection based on the highest number of detected discharges (nPulses), the highest pulse-to-noise ratio (PNR), and the lowest coefficient of variation of inter-spike interval (CoV_ISI_) during the plateau. (**Left panel**) Boxplots summarize the rate of agreement (RoA) values obtained across the 11 signals for the three tested criteria. (**Right panel**) Boxplots summarize the matched MU fraction obtained for the selection criteria.

As a second complementary analysis, three criteria for selecting among duplicate spike trains were compared: selection based on the lowest CoV_ISI_ during the plateau, the highest PNR, and the highest number of detected discharges. Figure 11 summarizes the effect of these criteria on the signal-level median RoA and the matched MU fraction between the automatic results and the manually edited spike trains. Selection based on the highest number of detected discharges showed the broadest distribution, with lower RoA and matched MU fraction values. In contrast, selection based on the highest PNR and the lowest CoV_ISI_ resulted in high median RoA values (0.98 and 0.97, respectively) and smaller IQRs across signals. Selection based on the highest PNR showed two lower outliers in the RoA distribution at 0.92 and 0.91, whereas selection based on the lowest CoV_ISI_ showed one lower outlier at 0.92. Overall, these results indicate that PNR- and CoV_ISI_-based selection provide more stable agreement than selection based only on the highest number of detected discharges.

### 4.2. Experimental HD-sEMG Signals in Healthy Volunteers

In the experimental study, 216 HD-sEMG signals (6 contractions × 4 sessions × 9 volunteers) were acquired from the TA during isometric trapezoidal muscle contractions. For four volunteers, 48 additional signals (3 contractions × 4 sessions for each volunteer) were acquired after electrode removal and repositioning to evaluate repeatability and the variability associated with electrode placement. The recorded HD-sEMG signals presented a peak-to-peak amplitude of 1.3 ± 0.6 mV in monopolar configuration and 497 ± 287 μV in the differential configuration. Their corresponding spectrum had frequencies between 8 ± 0.69 Hz–421 ± 38 Hz, with a maximum frequency peak around 43.6 Hz. These values were consistent with the characteristic physiological values of sEMG signals [11].

#### 4.2.1. Number of Detected MUs Across Sessions

Table 4 presents the mean and standard deviation of the number of detected MUs for each volunteer across the experimental sessions. The number of identified MUs remained stable across sessions within each volunteer, supporting the consistency of the proposed acquisition and analysis procedure.

#### 4.2.2. Physiological Properties of MUs

**Figure 12:**
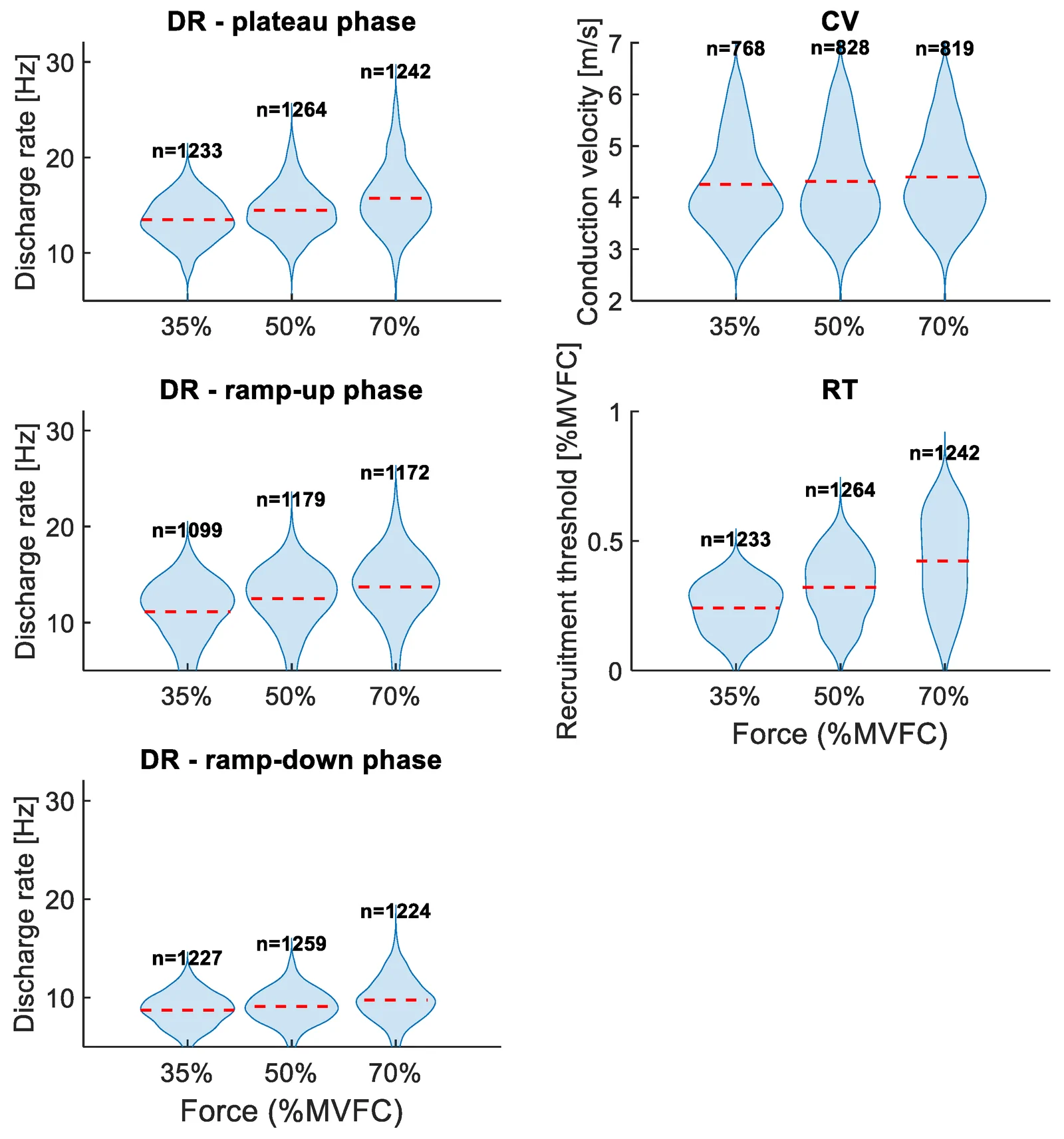
Violin plots showing the distribution of the estimate MU physiological parameters: discharge rate (DR), recruitment threshold (RT), and motor unit conduction velocity (MUCV). Parameters were grouped according to the contraction plateau force level (35%, 50% and 70%). An increasing trend with force level was observed for DR across the three contractions phases and for RT.

The physiological parameters of the MUs detected during the experimental protocol were estimated from the discharge times of the individual MUs. Figure 12 presents the distribution of the estimated parameters for the three target-force groups: 35%, 50% and 70% MVFC across all sessions and volunteers. The obtained MUCV values ranged from 2.47 to 6.49 m/s (mean 4.32 ± 0.86 m/s). During the plateau phase, DR values reached a maximum of 27.39 Hz (mean 14.57 ± 2.94 Hz).

The violin plots in Figure 12 illustrate the distribution of all identified MUs. Higher mean DR values across the three contraction phases, as well as higher normalized RT values, were observed for contractions performed at 50% and 70% MVFC than at 35% MVFC. To assess differences in the estimated MU parameters among the MU populations identified at each target-force level, separate linear mixed-effects models were fitted for each parameter, with target force as the fixed effect and random intercepts for volunteer, session nested within volunteer, and contraction.

For DR and normalized RT, significant differences were observed between contractions up to 50% MVFC and 70% MVFC compared with 35% MVFC. At 50% MVFC, the DR was significantly higher during ramp-up (β = 1.77, 95% CI = [1.28, 2.26], t = 7.09 and *p* < 0.001), ramp-down (β = 0.51, 95% CI = [0.24, 0.79], t = 3.73 and *p* < 0.001), and plateau (β = 1.21, 95% CI = [0.89, 1.52], t = 7.56 and *p* < 0.001), and the normalized RT was also significantly higher (β = 0.07, 95% CI = [0.05, 0.09], t = 7.11 and *p* < 0.001). For 70% MVFC relative to 35% MVFC, the same pattern was observed, with significant increases in DR during ramp-up (β = 2.98, 95% CI = [2.49, 3.47], t = 12.01 and *p* < 0.001), ramp-down (β = 1.20, 95% CI = [0.93, 1.47], t = 8.74 and *p* < 0.001), and plateau (β = 2.56, 95% CI = [2.24, 2.87], t = 16.07 and *p* < 0.001), together with a significant increase in the normalized RT (β = 0.18, 95% CI = [0.16, 0.20], t = 17.74 and *p* < 0.001).

For MUCV, the full hierarchical model resulted in a singular fit because the variance of the contraction-level random intercept was estimated as zero. The contraction random intercept was therefore removed, and the final model included random intercepts for volunteer and the session nested within the volunteer. Compared with the 35% MVFC group, MUCV was significantly higher at both 50% MVFC (β = 0.09, 95% CI = [0.02, 0.16], t = 2.45 and *p* = 0.014) and 70% MVFC (β = 0.17, 95% CI = [0.10, 0.24], t = 4.88 and *p* < 0.001).

#### 4.2.3. Within-Session Repeatability

**Figure 13:**
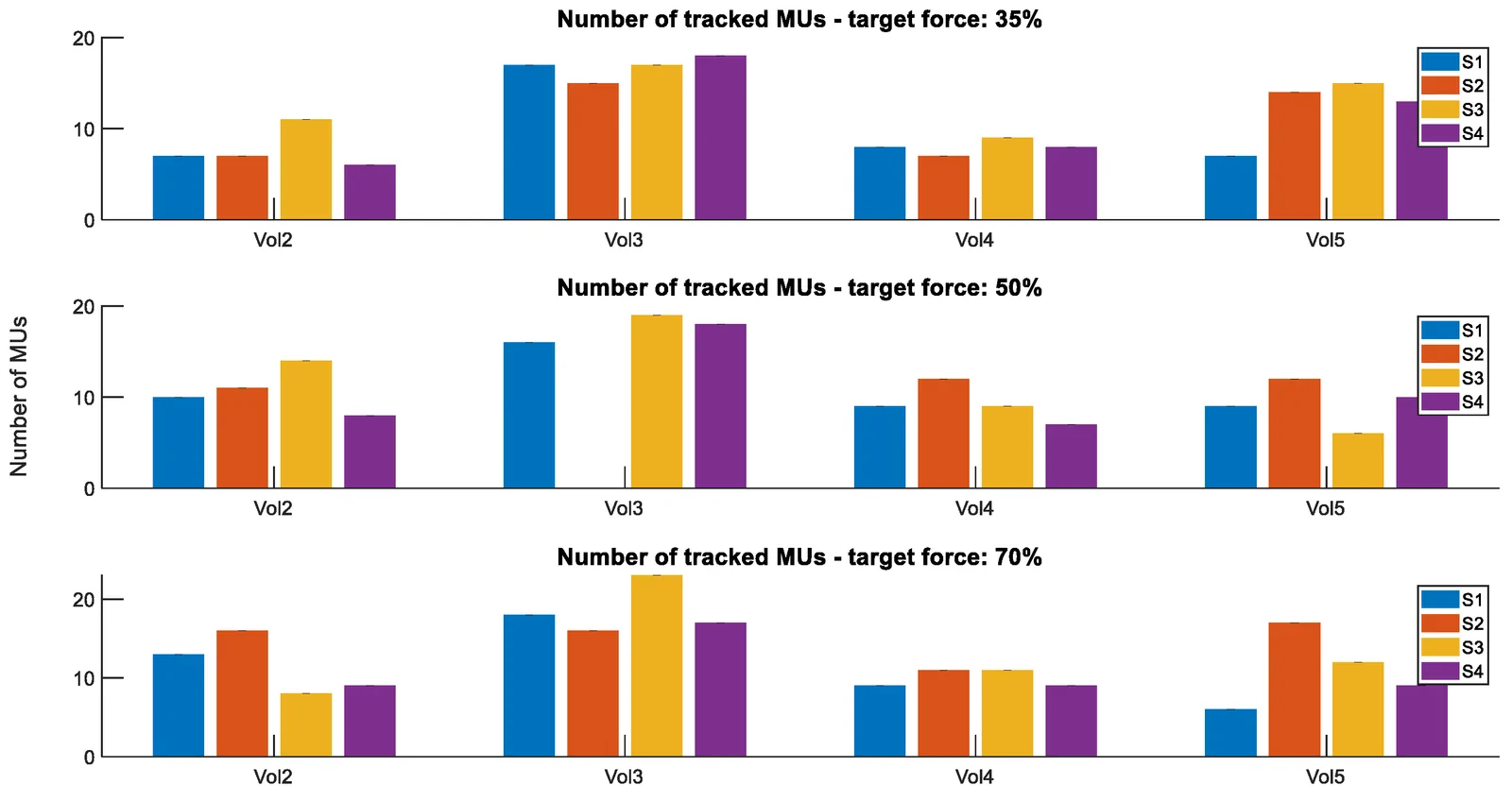
Number of MUs tracked after electrode removal and repositioning. MUs were tracked independently within each session using the normalized two-dimensional cross-correlation of their MUAP waveforms (threshold 0.7). Bar plots show the number of tracked MUs for each volunteer, session, and target force.

To assess within-session repeatability, the electrode matrix was removed and repositioned in four volunteers, and the MUs identified before and after this procedure were independently tracked within each session. For each force level, the pair of contractions with the highest number of tracked MUs was selected, and the corresponding MU parameters were compared. The number of tracked MUs varied across volunteers, with mean ± SD values of 10.00 ± 3.10 for Volunteer 2, 16.17 ± 5.47 for Volunteer 3, 9.08 ± 1.56 for Volunteer 4, and 10.83 ± 3.59 for Volunteer 5. Figure 13 shows the number of tracked MUs for each volunteer, session, and target force across the four sessions.

**Figure 14:**
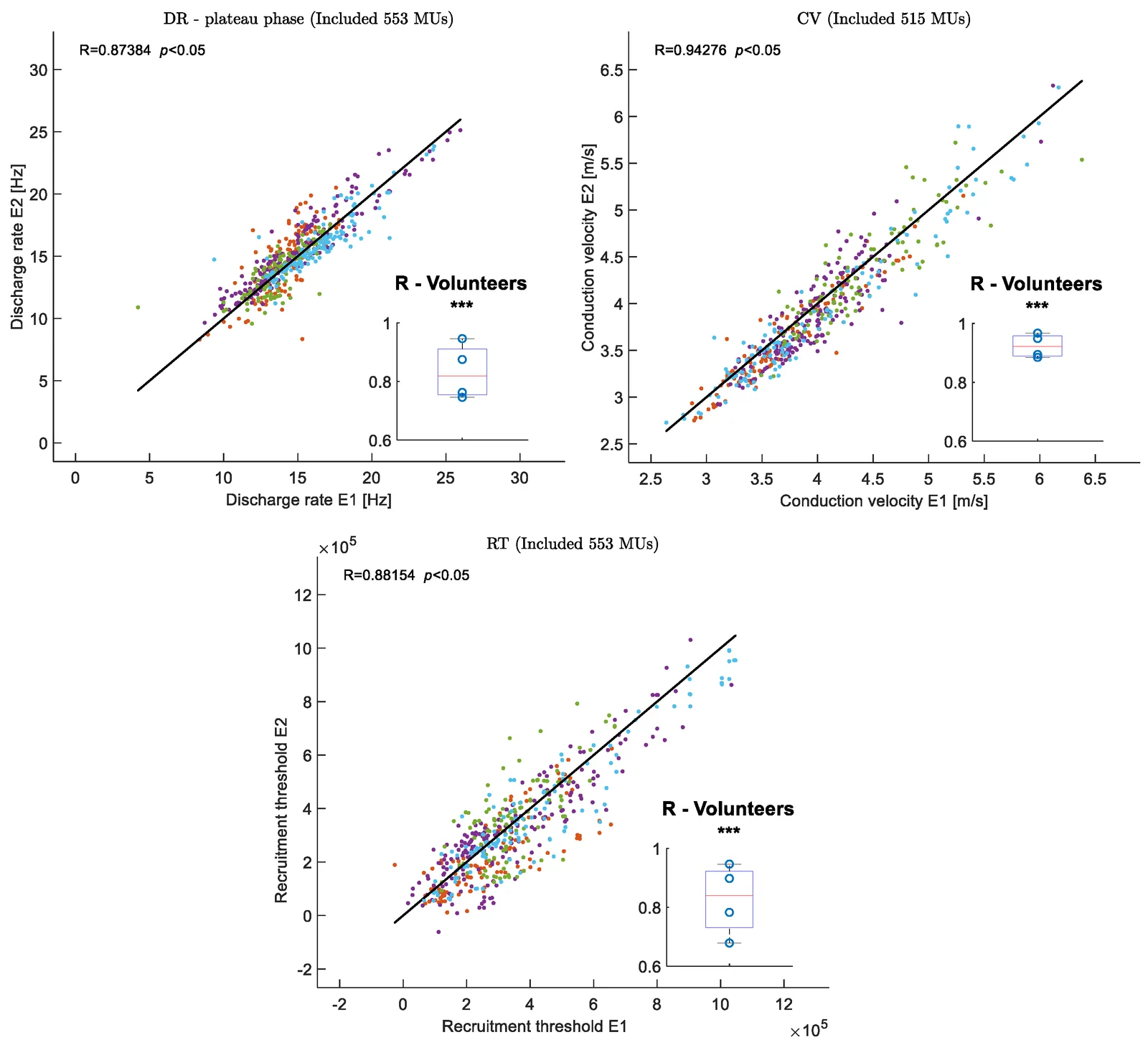
Comparison of the physiological parameters of the tracked MUs before and after electrode removal and repositioning (E1 vs. E2). Parameters include RT, DR and MUCV. The volunteers are coded by color. Significant Pearson correlations were observed for all physiological parameters in each volunteer and for all together. *** indicates p<0.001 for all volunteers.

Pearson correlation was used to compare the MU parameters before and after electrode removal and repositioning within each session (E1 and E2 in Figure 14). The correlations were computed using data pooled from all four sessions and all three target force levels. Table 5 summarizes the correlation coefficients for the tracked MUs. Significant correlations were obtained for all the analyzed physiological parameters, both when each volunteer was analyzed separately and when all volunteers were analyzed together (Figure 14). For Volunteers 3 and 5, all correlation coefficients were higher than 0.87. The only correlation below 0.7 was observed for RT in Volunteer 4 (r = 0.68, *p* < 0.001). Overall, these results indicate good within-session repeatability of the estimated MU parameters, even after electrode removal and repositioning.

### 4.3. Consistency of the Automatic Channel Selection Algorithm

To assess the consistency of the automatic channel-selection algorithm for MUCV estimation, MUCV values were compared before and after electrode repositioning in the tracked MUs. In a first analysis, for each tracked MU pair, MUCV was computed using the same set of electrode channels in both conditions. This set was chosen from the two automatically selected channel sets (before and after repositioning) as the one yielding the lower mse after waveform alignment. Under this condition, high correlations were obtained across the four volunteers (r = 0.95 *, 0.89 *, 0.88 *, 0.97 *; all *p* < 0.001).

In a second analysis, MUCV was computed using the channels selected independently by the automatic algorithm in each condition. In this case, the corresponding correlations were lower (r = 0.80 *, 0.83 *, 0.56 *, 0.88 *; all *p* < 0.001). These results suggest that the automatic algorithm identifies a similar MUAP propagation region before and after electrode repositioning, although not necessarily the exact same channels. This interpretation is supported by the small spatial differences between the independently selected channel sets: the median Euclidean distance ranged from 0.5 to 1.12 electrodes across volunteers, and the median column difference was 0 for three volunteers and 1 for one volunteer. The lower correlations obtained when channel selection was performed independently in each condition therefore indicate that, for some volunteers, MUCV estimation is sensitive to small differences in the selected channels within the propagation region.

### 4.4. Manual Validation of the Automatic Channel-Selection Algorithm

The automatic channel-selection algorithm was evaluated against manual channel selection in 78 MUs. Manual selection produced MUCV estimates within the physiological range of 2–6.5 m/s for 75 MUs (96.15%).

**Figure 15:**
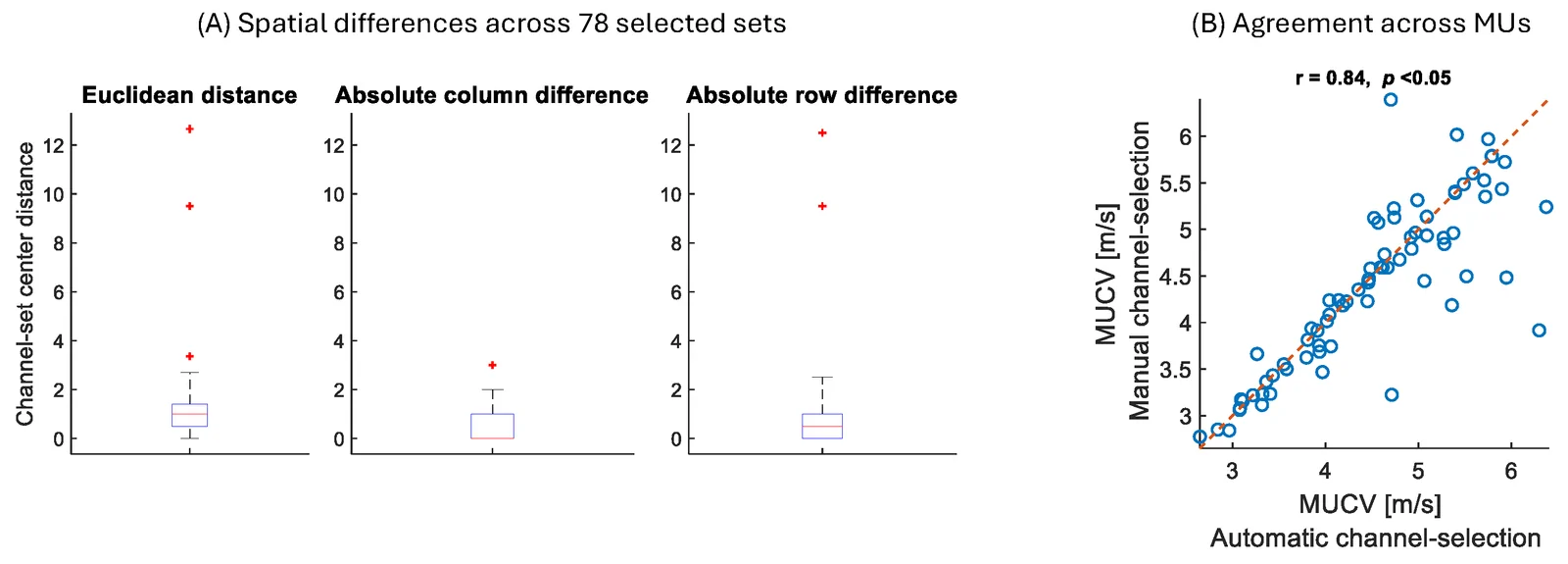
Comparison between automatic and manual channel selection for MUCV estimation. (**A**) Euclidean distance between the centers of the automatically and manually selected channel sets and absolute differences in their column and row positions. Distances are expressed as inter-electrode-distance units, with 1 IED corresponding to 4 mm. Red crosses indicate outliers. (**B**) Relationship between MUCV estimates obtained using the automatically and manually selected channel sets after excluding the three MUCV values outside the physiological range of 2–6.5 m/s (*n* = 75). The dashed lines indicate the line of identity. r: Pearson correlation coefficient, MUCV: motor unit conduction velocity.

Figure 15A summarizes the spatial differences between the automatically and manually selected channel sets. The Euclidean distance between the channel-set centers was concentrated at low values, with an upper whisker of 2.69 IEDs (10.8 mm), and three outliers. The absolute difference in column position had an upper whisker of 2 IEDs and a maximum of 3 IEDs (12 mm). The absolute difference in row position had an upper whisker of 2.5 IEDs, with two outliers and a maximum of 12.5 IEDs (50 mm).

When all 78 MUs were considered, the MUCV estimates obtained using the automatically and manually selected channel sets showed a Pearson correlation of (r = 0.36, *p* < 0.05). The median difference (automatic-manual channel selection) was 0 m/s (IQR = 0.23 m/s), with a mean absolute difference of 0.68 m/s. After excluding the three MUCV estimates outside the physiological range, the correlation increased to (r = 0.84, *p* < 0.05), as shown in Figure 15B, while the median difference remained 0 m/s (IQR = 0.22 m/s) and the mean absolute difference decreased to 0.28 m/s.

### 4.5. Runtime Assessment

For the public database, the complete decomposition and automated source-validation procedure required 28.22 min to process the 11 signals.

For the experimental dataset, the complete manual channel-selection procedure, including calculation of the adjacent-channel cross-correlation and MUCV estimation, required 128.75 min for the 78 MUs included in the manual validation. This duration included approximately 46 s per MU for data loading and MUAP waveform display. The complete automatic parameter-estimation procedure required 329.59 min to process all MUs from the 276 experimental signals, including RT, DR, and MUCV estimation and automatic channel selection. These measurements represent the active operator time for a subset of MUs and the computational runtime for the complete experimental dataset, respectively, and therefore characterize manual analysis effort and computational demand rather than providing a direct comparison between human and computer processing time.

## 5. Discussion

This study presented and validated an automated pipeline for MU analysis from HD-sEMG signals acquired during isometric trapezoidal contractions. The main contribution was the automation of stages that still often depend on expert manual intervention, particularly HD-sEMG decomposition through integration of the BSS algorithm with automated source validation and spike-train selection, as well as automatic channel selection for MUCV estimation.

The BSS algorithm was applied to the entire recording using 200 source-estimation iterations, aiming to identify up to 200 candidate sources and their corresponding spike trains. These candidate spike trains were then processed through an ordered automated procedure based on the expected discharge behavior during the stable portion of the plateau. Candidates with implausibly high discharge rates or an insufficient number of discharges were first excluded. Duplicate spike trains were then identified, and one spike train from each duplicate group was retained using the CoV_ISI_ metric evaluated during the plateau. This sequence prevented non-physiological candidates from influencing duplicate resolution and used the expected low inter-spike interval variability during the plateau as the basis for source validation and final spike-train selection. Importantly, the plateau was used only to apply the exclusion, duplicate-identification, and selection criteria. For each retained spike train, all discharges identified across the full recording were retained for subsequent MU parameter estimation.

Validation on a public database showed high agreement between the proposed automated procedure and manually edited spike trains for successfully matched MUs, while the experimental evaluation in healthy volunteers demonstrated that the pipeline provides physiologically plausible MU parameters with good within-session repeatability.

Across the dataset, 106 of 135 reference MUs were matched, and 96 of the matched MUs showed median agreement above 0.9, with an overall median RoA of 0.98. These agreement values apply only to the matched MUs. Not all MUs were matched across approaches: 30 reference MUs were not identified, whereas the automated procedure detected 47 extra MUs. These additional MUs are not necessarily false positives or the result of over-splitting, because the proposed method includes duplicate removal and spike-train quality filtering based on PNR. In addition, the public database itself shows that manual editing reduces the final number of retained MUs and varies across operators. Differences in decomposition parameters, including the number of iterations, may also have contributed to the observed mismatch.

Complementary analyses further supported the robustness of the proposed spike-train selection procedure. Plateau duration mainly affected agreement with the reference manually edited spike trains, with longer stable segments providing more reliable CoV_ISI_ estimates. In practice, the agreement obtained with a plateau duration of 7 s was comparable to that obtained with 10 s while remaining suitable for the present protocol because it avoided transient effects associated with force stabilization. The CoV_ISI_ threshold primarily affected MU retention: more permissive values increased the number of matched MUs but also admitted a small number of lower-agreement units. By contrast, the minimum number of spikes and the upper discharge-rate limit did not show significant effects on the evaluated metrics. Comparison of duplicate-selection criteria showed that CoV_ISI_-based and PNR-based selection resulted in similarly high agreement values, whereas selection based only on the highest number of detected discharges yielded the lowest matched fraction and the highest variability in agreement quality. Because the aim of the present study was to validate a procedure based on the expected discharge regularity during the plateau phase, CoV_ISI_ was retained as the default criterion. Nevertheless, the good performance obtained using the PNR criterion suggests that it may be a useful alternative, particularly in pathological conditions in which discharge regularity may be less preserved.

In the experimental evaluation, 276 HD-sEMG signals were acquired from the TA muscle of nine healthy volunteers during four experimental sessions conducted over one month. First, a protocol for standardized electrode placement was proposed based on the results of a preliminary test that identified the region above the IZ as the optimal electrode placement over the TA muscle. This test highlighted the importance of identifying the location of the IZ and of placing the electrode matrix relative to it, because this placement has a significant effect on the number of identifiable MUs. The HD-sEMG signals acquired with our custom-built device showed typical signal characteristics. The BSS algorithm and the automated procedure were used to decompose the experimental signals, and the number of detected MUs remained consistent across sessions within volunteers, supporting the stability of MU identification across repeated measurements.

Some limitations should be acknowledged. The experimental evaluation was performed only in healthy volunteers. In clinical populations, even during isometric trapezoidal contractions, a stable plateau phase may not always be achieved. Under these conditions, CoV_ISI_ alone may be less suitable and may need to be complemented by PNR-based criteria for duplicate spike-train selection.

The experimental evaluation in healthy volunteers showed that the complete pipeline produced physiologically plausible MU parameter estimates. The estimated MUCV values were consistent with previously reported experimental values for MUs identified in the TA muscle [37]. Similarly, plateau DR values were within the physiological ranges reported for submaximal isometric contractions [20,36].

The linear mixed-effects models showed that the mean DR values of the sampled MU populations were higher during the ramp-up, plateau, and ramp-down phases at both 50% and 70% MVFC than at 35% MVFC. The findings for the plateau phase are consistent with a previous study reporting higher mean MU firing rates at higher force levels during steady-state isometric contractions of the TA [36]. Normalized RT and MUCV were also significantly higher at both 50% and 70% MVFC than at 35% MVFC. Because individual MUs were not tracked across force levels, these findings represent differences among the sampled MU populations rather than changes within the same MUs. Accordingly, the higher normalized RT values indicate that the populations sampled during higher-force contractions included MUs recruited at higher thresholds. The higher MUCV values observed in the proximal portion of the TA may similarly reflect the recruitment of higher-threshold MUs. This interpretation is consistent with previous findings showing a positive association between RT and MUCV in the distal portion of the TA [8], although the difference in electrode placement limits direct comparison. Together, the physiological ranges of the estimated parameters and the observed force-related differences support the physiological plausibility of the estimates obtained using the automated pipeline.

The within-session repeatability analysis provided additional support for the stability of the pipeline, even after intentional removal and repositioning of the electrode matrix. Significant correlations were observed for all physiological parameters before and after electrode removal and repositioning. Although RT in one volunteer showed a lower correlation, the overall results suggest that the estimated parameters are robust across repeated contractions within the same session. These findings support the within-session repeatability of the proposed methodology.

The automatic channel-selection procedure for MUCV estimation also showed good consistency. High correlations were obtained when the same selected channel set was used before and after repositioning, whereas correlations were lower when the electrodes were selected independently in each condition. This suggests that the proposed image-processing approach identifies a similar MUAP propagation region across repeated measurements, although the final MUCV estimate may still be sensitive to small local differences in channel selection. This interpretation is supported by the low median Euclidean distance and median column difference between the selected channel sets.

The comparison with manual channel selection provided complementary support for the automatic procedure. The automatically and manually selected channel sets showed small spatial differences overall, although larger differences occurred in 3 of the 78 evaluated MUs. After excluding the MUCV estimates outside the physiological range, the automatic and manual MUCV estimates were strongly correlated, with a median difference of 0 m/s, and a mean absolute difference of 0.28 m/s. Together, these results suggest that the automatic channel-selection procedure identifies MUAP propagation regions similar to those selected manually in most cases and produces comparable MUCV estimates, while also indicating that MUCV estimation may remain sensitive to local differences in channel selection.

The runtime assessment quantified the practical demands of the analysis. The public dataset was processed automatically in 28.22 min, compared with more than 2 h of manual editing reported for approximately 100 MUs [16]. In the experimental dataset, manual channel selection and MUCV estimation required 128.75 min for 78 MUs compared to 329.59 min to process all MUs from the 276 experimental signals. Because these values compare the active operator time with the computational runtime, they should not be interpreted as a direct comparison of processing efficiency. Instead, they provide complementary information on the manual and computational time associated with the analysis stages.

## 6. Conclusions

The proposed pipeline enables automated MU analysis of HD-sEMG signals acquired from healthy volunteers during isometric trapezoidal contractions. The BSS algorithm combined with automated source-validation and spike-train selection procedure showed high agreement with manually edited spike trains for successfully matched MUs. In healthy volunteers, the complete pipeline also produced physiologically plausible MU parameter estimates. In most cases, the automatic channel-selection algorithm selected channel sets that were spatially close to those selected manually, and the resulting MUCV estimates were highly correlated after excluding manual estimates outside the physiological range. In addition, good within-session repeatability of MU properties was observed after electrode removal and repositioning. The key next steps are to extend the evaluation to a larger number of volunteers, assess the repeatability across sessions. and validate the pipeline in clinical populations.

## Figures and Tables

**Table 1:** Comparison of the HD-M-sEMG device specifications with reference values reported in the literature [11]. IED: inter-electrode distance. CMRR: common mode rejection ratio. $Z_{i}$: input impedance. LPF: low-pass filter. HPF: high-pass filter. A/D: analog-to-digital.

Parameters	Theoretical Values	HD-M-sEMG Device Values
Detection		
Electrode material	Non-polarizable electrodes	Silver-coated electrodes
Electrode size	Diameter < 3–5 mm	1.95 mm
IED	<5–10 mm	4 mm
Conditioning		
CMRR	90–110 dB at 50 Hz	82 dB at 50 Hz
$Z_{i}$	>80 MΩ at 50 Hz	1300 MΩ at 10 Hz
LPF cut-off frequency	400–900 Hz	800 Hz
HPF cut-off frequency	5–30 Hz	8 Hz
Sampling and A/D Conversion		
Sampling frequency	>1.5 kHz	3.0706 kHz
Resolution	0.5–1.0 μV	0.195 μV
A/D resolution	14–16 bits	16 bits

**Table 2:** Decomposition performance of our proposed automated procedure using the default parameter configuration. For each signal in the database, the table presents the range of MU spike trains available in the database across operators, the number identified by the automatic procedure, the number of matched MUs, the number of missed reference MUs, and the number of extra automatic MUs. The last row presents totals across signals. MVFC: maximal voluntary force contraction. MU: motor unit. TA: tibialis anterior. GM: gastrocnemius medialis. GL: gastrocnemius lateralis.

Muscle	%MVFC	Manual MUs Across Operators (Min–Max)	Automatic MUs	Matched MUs	Missed Reference MUs	Extra Automatic MUs
TA	35	19–21	29	19	2	10
TA	50	12–15	16	8	7	8
TA	70	6–8	18	8	0	10
GM	10	16–18	12	12	7	0
GM	30	24–28	29	23	5	6
GM	50	15–16	13	11	5	2
GM	70	5–6	14	6	0	8
GL	10	4–4	4	4	0	0
GL	30	8–10	10	9	1	1
GL	50	4–4	5	3	1	2
GL	70	4–5	3	3	2	0
Total		117–135	153	106	30	47

**Table 3:** Agreement between the automatically selected spike trains and the manually edited spike trains in the reference database. For each matched MU, the RoA across operators was first summarized using the median and IQR. The table presents, for each signal, the median and IQR for these MU-level values. MVFC: maximal voluntary force contraction. MU: motor unit. RoA: rate of agreement. IQR: interquartile range. TA: tibialis anterior. GM: gastrocnemius medialis. GL: gastrocnemius lateralis.

Muscle	%MVFC	Median of MU-Level Median RoA	IQR of MU-Level Median RoA	Median of MU-Level Operator IQR	IQR of MU-Level Operator IQR
TA	35	0.995	0.007	0.000	0.005
TA	50	0.988	0.010	0.002	0.003
TA	70	0.969	0.097	0.006	0.008
GM	10	0.984	0.019	0.002	0.005
GM	30	0.971	0.042	0.002	0.005
GM	50	0.974	0.038	0.002	0.006
GM	70	0.918	0.085	0.005	0.007
GL	10	0.986	0.008	0.005	0.004
GL	30	0.957	0.087	0.002	0.004
GL	50	0.985	0.104	0.002	0.007
GL	70	0.945	0.015	0.005	0.005

**Table 4:** Number of MUs estimated from the acquired HD-sEMG signals for each volunteer in session 1, session 2 (1 h), session 3 (15 days), and session 4 (30 days). MU: motor unit. HD-sEMG: high-density surface electromyography. nVol: Volunteer identifier.

nVol	Session 1	Session 2 (1 h)	Session 3 (15 Days)	Session 4 (30 Days)
1	10.00 ± 3.08	9.22 ± 3.83	10.22 ± 1.79	10.89 ± 1.27
2	20.56 ± 6.98	24.12 ± 6.06	23.22 ± 5.74	17.33 ± 9.66
3	35.22 ± 2.86	32.00 ± 5.76	38.33 ± 5.20	30.00 ± 5.22
4	14.44 ± 1.42	14.33 ± 4.12	14.44 ± 1.59	11.67 ± 2.18
5	13.33 ± 3.97	16.56 ± 2.07	13.33 ± 3.46	15.11 ± 1.45
6	17.75 ± 1.49	20.40 ± 1.34	19.50 ± 3.08	19.17 ± 1.33
7	7.83 ± 1.94	5.33 ± 1.75	9.17 ± 3.66	7.33 ± 2.42
8	24.67 ± 2.16	25.00 ± 4.73	27.67 ± 2.25	22.33 ± 5.92
9	22.50 ± 2.26	17.00 ± 6.23	17.83 ± 3.54	14.50 ± 4.76

**Table 5:** Pearson correlations between tracked MUs within sessions. RT: recruitment threshold. DR_P: mean discharge rate during the plateau. MUCV: motor unit conduction velocity. *r*: Pearson correlation coefficient. nMUs: number of MUs included in the correlation analysis. * denotes statistical significance (p<0.05).

	Vol2		Vol3		Vol4		Vol5	
	*r*	nMUs	*r*	nMUs	*r*	nMUs	*r*	nMUs
RT	0.78 *	120	0.90 *	194	0.68 *	109	0.95 *	130
DR_P	0.75 *	120	0.95 *	194	0.76 *	109	0.87 *	130
MUCV	0.95 *	113	0.89 *	175	0.88 *	102	0.97 *	125

## Data Availability

The data obtained from clinical investigation participants are not publicly available due to privacy, confidentiality, and data protection restrictions. Data may be made available from the corresponding author upon reasonable request.

## Abbreviations

The following abbreviations are used in this manuscript:

A/D	analog-to-digital
AP	action potential
BSS	blind source separation
CMRR	common mode rejection ratio
${\mathrm{CoV}}_{\mathrm{ISI}}$	coefficient of variation of inter-spike interval
DR	discharge rate
GL	gastrocnemius lateralis
GM	gastrocnemius medialis
HD-M-sEMG	high-density modular surface electromyography
HD-sEMG	high-density surface electromyography
IED	inter-electrode distance
IQR	interquartile range
ISI	inter-spike interval
IZ	innervation zone
LAL	anatomical landmark length
LME	linear mixed-effects model
LSD	longitudinal single differential
MFCV	muscle fiber conduction velocity
mse	mean square error
MU	motor unit
MUAP	motor unit action potential
MUCV	motor unit conduction velocity
MVFC	maximal voluntary force contraction
PNR	pulse-to-noise ratio
RoA	rate of agreement
ROI	regions of interest
RT	recruitment threshold
sEMG	surface electromyography
STA	spike-triggered averaging
SUPSI	University of Applied Sciences and Arts of Southern Switzerland
TA	tibialis anterior
USI	Universita della Svizzera italiana

## Appendix A

This section describes the preliminary test performed to identify the optimal electrode placement over the tibialis anterior (TA) muscle. Five healthy volunteers participated in this preliminary test. According to the literature [1], the innervation zone (IZ) is located, on average, at 34% of the anatomical landmark length, defined as the line between the tibial tuberosity and the intermalleolar line [1]. The electrode matrix was initially placed at 34% of this anatomical landmark length and then moved along the vertical axis until identifying minimum HD-sEMG signal amplitude and signal phase inversion (Figure 7). These characteristics are representative of the HD-sEMG signals acquired from the IZ when visualized in LSD configuration (Section 2.2.2, for review, see [8]). The HD-sEMG signals were filtered using a band-pass filter (20–500 Hz) and a notch filter at 50 Hz (Section 2.2.1). The median IZ location across the five volunteers was 46% of the anatomical landmark length.

After identifying the IZ, HD-sEMG signals were acquired from the TA muscle of the five healthy volunteers during trapezoidal contractions up to target forces of 35% and 50% of MVFC (two repetitions for each target force), following the procedures described in Section 3.2. The electrode matrix of the HD-M-sEMG device (Figure 6) was placed parallel to the direction of AP propagation, with its center row positioned close to the distal IZ. The HD-sEMG signals were then decomposed separately for three electrode positions relative to the IZ (above, centered over, and below the IZ) using the implementation of the decomposition algorithm followed by the proposed automated spike-train selection and validation procedure described in Section 2.3.

A three-way repeated measures analysis of variance (ANOVA, *p* < 0.05) was used to analyze the number of identified MUs, with muscle region (3 levels), target force (2 levels), and repetition (2 levels) as factors. Statistical significance was set at *p* < 0.05. This statistical analysis was performed using IBM SPSS Statistics version 27. A significant main effect of the muscle region was found (F = 9.154, *p* = 0.034), with the highest number of identified MUs observed above the distal IZ. MUAP propagation was observed in all three regions.
